# Amsacrine Derivatives Selectively Inhibit Mycobacterial Topoisomerase I (TopA), Impair *M. smegmatis* Growth and Disturb Chromosome Replication

**DOI:** 10.3389/fmicb.2018.01592

**Published:** 2018-07-17

**Authors:** Marcin J. Szafran, Marta Kołodziej, Patrycja Skut, Brahmam Medapi, Agnieszka Domagała, Damian Trojanowski, Jolanta Zakrzewska-Czerwińska, Dharmarajan Sriram, Dagmara Jakimowicz

**Affiliations:** ^1^Laboratory of Molecular Microbiology, Faculty of Biotechnology, University of Wroclaw, Wroclaw, Poland; ^2^Department of Pharmacy, Birla Institute of Technology and Science-Pilani, Hyderabad, India; ^3^Faculty of Chemistry, Wroclaw University of Technology, Wroclaw, Poland; ^4^Laboratory of Microbiology, Ludwik Hirszfeld Institute of Immunology and Experimental Therapy, Polish Academy of Sciences, Wroclaw, Poland

**Keywords:** TopA, topoisomerase, *Mycobacterium*, TLMM, DNA relaxation

## Abstract

Amsacrine, which inhibits eukaryotic type II topoisomerase via DNA intercalation and stabilization of the cleavable topoisomerase-DNA complex, promotes DNA damage and eventually cell death. Amsacrine has also been shown to inhibit structurally distinct bacterial type I topoisomerases (TopAs), including mycobacterial TopA, the only and essential topoisomerase I in *Mycobacterium tuberculosis*. Here, we describe the modifications of an amsacrine sulfonamide moiety that presumably interacts with mycobacterial TopA, which notably increased the enzyme inhibition and drug selectivity *in vivo*. To analyse the effects of amsacrine and its derivatives treatment on cell cycle, we used time-lapse fluorescence microscopy (TLMM) and fusion of the β-subunit of DNA polymerase III with enhanced green fluorescence protein (DnaN-EGFP). We determined that treatment with amsacrine and its derivatives increased the number of DnaN-EGFP complexes and/or prolonged the time of chromosome replication and cell cycle notably. The analysis of TopA depletion strain confirmed that lowering TopA level results in similar disturbances of chromosome replication. In summary, since TopA is crucial for mycobacterial cell viability, the compounds targeting the enzyme disturbed the cell cycle and thus may constitute a new class of anti-tuberculosis drugs.

## Introduction

The genus *Mycobacterium* encompasses Gram-positive, rod-shaped bacilli, including the relatively fast-growing (division time 2–3 h) saprophyte *Mycobacterium smegmatis* and the slow-growing (division time 24 h) pathogen *Mycobacterium tuberculosis*, the causative agent of tuberculosis. According to recent WHO data (WHO, 2016), over 10 million new tuberculosis cases are reported each year, and among them, almost 0.5 million infections are caused by multidrug-resistant forms (MDRs) of *M. tuberculosis*. The resistance of pathogenic bacteria to already known antibiotics justifies efforts to identify new chemical compounds and/or putative targets for anti-mycobacterial treatments. Because of the low homology between bacterial and eukaryotic topoisomerases, they are regarded as important antibiotic targets.

Topoisomerases are a broad group of enzymes that are crucial for cell survival. They regulate the number of contacts between two DNA strands in a double helix through transient breaking and re-joining of phosphodiester bond, thereby maintaining the optimal chromosomal supercoiling (Champoux, 2001; Viard and de la Tour, 2007; Forterre and Gadelle, 2009). Their function is particularly important for removal of topological tension generated by unwinding the DNA double helix during movement of the replication and transcription machinery. The proper movement of the replication complex is enabled by the action of gyrase (a type II topoisomerase), which is responsible for removing the excess positive DNA supercoils in front of the complex in an ATP-hydrolysis-dependent manner (Khodursky et al., 2000). Topoisomerase I (TopoI, TopA, type I topoisomerase), as the enzyme that removes excess of negative supercoils, is not the component of replication forks; however, TopA activity has been shown to prevent uncontrolled DNA replication from R-loops (Wimberly et al., 2013; Martel et al., 2015) and thus may indirectly affect chromosome replication. Moreover, TopA facilitates the progression of transcription by removing R-loops (RNA-DNA hybrids). Therefore, the inhibition of the activity of each topoisomerase should affect not only global supercoiling but also the progression of transcription and replication.

In bacterial cells, the replication forks can be visualized by the fusion of DNA polymerase III subunits (e.g., DnaN) with a fluorescent protein (e.g., enhanced green fluorescence protein, EGFP). Chromosome replication starts at a specific site, *oriC* (*the origin of chromosome replication*), by assembly of the multiprotein replication complex (replisome) and proceeds bidirectionally until it reaches the termination region (*ter*). Depending on the bacterial genus, the replisome may remain positioned in the mid-cell proximity (*Bacillus subtili*s; Berkmen and Grossman, 2006; Wang et al., 2014) or may change its position during replication moving from mid-cell toward cell pole (*Escherichia coli* Reyes-Lamothe et al., 2008). In *M. smegmatis*, shortly after initiation of chromosome replication, a single DnaN-EGFP focus splits into two foci, which remain separated by a short distance (10–20% of the cell length) and positioned slightly asymmetrically near the mid-cell until their disappearance when replication is completed within 120–130 min (Santi and McKinney, 2015; Trojanowski et al., 2015). Since the progression of chromosome replication is dependent not only on replication machinery but also on accessory proteins including topoisomerases, the inhibition of topoisomerase activity should be reflected in altered dynamics of replication.

Although both types of topoisomerases are valuable targets for antibacterial and anticancer therapies because of their involvement in replication and transcription, the vast majority of already known inhibitors target eukaryotic and bacterial type II topoisomerase. These compounds exhibit different modes of action, including trapping of the topoisomerase-DNA covalent complex (amsacrine, anthracyclines, and quinolones), blocking of ATP binding (novobiocin) or inhibiting completion of the enzyme catalytic cycle (merbarone and dexrazoxane) (for more information, see two excellent reviews; Bailly, 2012; Pommier, 2013). Recent studies have shown that amsacrine, an eukaryotic topoisomerase II inhibitor used widely in lymphocytic and non-lymphocytic leukaemias (Jehn and Heinemann, 1991), also inhibits the activity of bacterial type I topoisomerase (TopA) (Godbole et al., 2014). The aminoacridine ring in amsacrine intercalates with DNA, whereas its sulfonamide side chain is suggested to interact with topoisomerases (both bacterial TopA and eukaryotic Topo II) (Denny and Wakelin, 1986; Zwelling et al., 1992; Ketron et al., 2012; Jangir et al., 2013). Amsacrine has been shown to effectively inhibit the activity of *E. coli* TopA (*Ec*TopA) and TopA homologs from *M. smegmatis* (*Ms*TopA) and *M. tuberculosis* (*Mt*TopA) and the model of its action has been proposed (Godbole et al., 2014). It also inhibits cell culture growth; however, the exact consequence of TopA inhibition in bacterial cells remains unclear. In contrast to *E. coli*, TopA in mycobacteria is the only and essential type I topoisomerase. Moreover, mycobacterial TopAs (as well as another previously studied actinobacterial topoisomerase I—*Streptomyces coelicolor* TopA; Szafran et al., 2014, 2016; Strzalka et al., 2017) exhibit unusually high processivity and differ remarkably from other bacterial homologs in that the long C-terminal domain lacks Zn^2+^ finger motifs (Bhaduri et al., 1998). Presumably, as in the related *Streptomyces* TopA, the high processivity of mycobacterial TopA is provided by the C-terminal lysine repeats, which are also required for DNA binding (Ahmed et al., 2013; Strzalka et al., 2017). In summary, because of the indispensability of the unique-to-mycobacteria type I topoisomerase, its inhibition may be a powerful strategy for designing efficient and selective therapies against mycobacterial infections.

Here, we have analyzed the influence of amsacrine and four of its selected derivatives on TopA activity *in vitro* and on *M. smegmatis* growth. We showed that modifications of amsacrine lead to increase in inhibition efficiency and higher selectivity for mycobacterial TopA protein. Moreover, we used DnaN-EGFP fusion to observe the influence of the amsacrine and its derivative on DNA replication and cell cycle and detected the similar disturbances as resulting from TopA depletion.

## Materials and methods

### Synthesis of the amsacrine derivatives

To synthetize the amsacrine derivatives, 2-chlorobenzoic acid was reacted with the corresponding aniline in the presence of copper powder in DMF, yielding diphenylamine derivatives. Upon treatment with POCl_3_, these compounds afforded 9-chloroacridine derivatives, which, after treatment with 2-methoxy-4-nitroaniline in the presence of p-toluenesulfonic acid followed by reduction with Fe-HCl, provided amino derivatives. Treatment of these amino with various aryl isocyanates, aryl isothiocyanates and alkyl/aryl sulfonyl chlorides yielded the corresponding urea, thiourea and sulfonamide derivatives, respectively (for more details, see the Supplementary Information).

### Cloning and purification of the *Ms*TopA protein

To purify the *Ms*TopA protein, the *topA* gene from *M. smegmatis* was amplified (using Ms*topA*_NdeI_FW and Ms*topA*_BamHI_RV oligonucleotides, Table S1) and subsequently cloned into pET-28a(+) expression vector (Invitrogen). The overproduction of His-tagged *Ms*TopA protein was performed in *E. coli* BL21 strain in the presence of 0.5 mM isopropyl β-D-1-thiogalactopyranoside (IPTG) for 3 h at 37°C. The *Ms*TopA protein was purified using a 5 ml HisTrap HP column (GE Healthcare, Life Sciences) and was subsequently tested for nuclease contamination according to the procedure described previously (Szafran et al., 2013).

### Topoisomerase I activity assays

The amsacrine derivatives were stored at 4°C as 20 mM stock solutions in DMSO. Shortly before the activity assays, the inhibitors were diluted in DMSO to reach working concentration (0.15–0.6 mM). An 18 μl mixture containing 120 ng *Ms*TopA, 25 mM NaH_2_PO_4_ at pH 8.0, 150 mM NaCl, 5% glycerol, 10 mM MgCl_2_ and a tested inhibitor in a final concentration of 15, 30, or 60 μM was incubated for 30 min on ice. To start the reaction, 2.0 μl (140 ng) of negatively supercoiled pUC19 plasmid were added to the mixture and incubated for 15 min at 37°C in the presence of 60 nM *Ms*TopA. The reaction was stopped by adding 1.5 μl of 0.5 M EDTA. DNA was resolved in 0.8% agarose in TAE buffer at 140 V for 3 h at room temperature. Topoisomers distribution was visualized by ethidium bromide staining for 30 min at room temperature. Subsequently, the fluorescence signal of supercoiled plasmid and the relaxed topoisomers was calculated using ImageJ Fiji suite (http://fiji.sc/Fiji). To determine the efficiency of *Ms*TopA inhibition, we calculated the fraction of total plasmid that remained supercoiled after the reaction.

In the inhibition assays used to test the effect of inhibitor binding to the enzyme, DNA or the enzyme-DNA complex (PI—protein-inhibitor binding, NI—nucleic acid-inhibitor binding, or PNI—protein-nucleic acid binding) the complete reaction mixtures (as above) without magnesium ions and one of the reaction components (substrate, enzyme or inhibitor in the PI, NI, and PNI assays, respectively) were preincubated for 30 min on ice. Subsequently, the reactions were started by adding magnesium chloride and the missing component: negatively supercoiled pUC19 plasmid, *Ms*TopA protein or the particular inhibitors. The reactions were performed for 5 min at 37°C and stopped by adding 1.5 μl of 0.5 M EDTA. Topoisomers were resolved and analyzed according to the procedure described above.

### Bacterial strains

All bacterial strain used in this study are listed in Table 1. For details of strains construction, see the Supplementary Information and Table S1.

**Table 1:** Strains used in this study.

**Strain**	**Relevant genotype or description**	**Source**
***E. coli***		
DH5α	*supE44*Δ*lacU169(*φ*80 lacZ*Δ*M15)hsdR17 recA1 endA1 gyrA96 thi-1 relA1*	Lab stock
BL21	B F^−^*dcm ompT hsdS*(rB^−^mB^−^) *gal*	Lab stock
***S. venezuelae***		
ATTC10712	Wild-type/laboratory strain	Lab stock
***M. smegmatis***		
mc^2^155	Laboratory strain	Lab stock
JH01	*M. smegmatis* mc^2^155 enzyme or DNA rathe*dnaN-egfp*	Trojanowski et al., 2015
MSZ1	*M. smegmatis* mc^2^155 *topA*::*apra* pMV306-Ms*topA*	This study
MSZ2	*M. smegmatis* mc^2^155 *topA*::*apra* pMV306-*Mt*topA	This study
PS17	*M. smegmatis* mc^2^ *ΔtopA, dnaN-egfp, parB*-*mcherry*, attBL5::pTC-28S15-0X psmyc*tetRrev*pmyc1*topA*	This study
PS29	*M. smegmatis* mc^2^, *dnaN-egfp, parB*-*mcherry*, attBL5::pTC-28S15-0X psmyc*tetRrev*pmyc1	This study

### Growth analysis

All *M. smegmatis* strains were grown in Middlebrook 7H9 medium (Difco) supplemented with 10% OADC (Becton, Dickinson and Company) and 0.05% Tween-80 at 37°C. *Streptomyces venezuelae* was grown in MYM medium supplemented with Trace Element Solution (TES) (Kieser et al., 2000) at 30°C. *E. coli* strains were grown in Luria-Bertani medium at 37°C. For growth analyses, *M. smegmatis* and *E. coli* strains were grown to the mid log-phase. Subsequently, the bacterial cultures were diluted to an optical density (OD600) of 0.01 (*M. smegmatis*) or 0.001 (*E. coli*) using media dedicated to the particular species. *S. venezuelae* cultures were inoculated with spores diluted to OD600 2^*^10^−5^ with MYM medium. To determine the growth rate, the optical density measurements were taken in 20 min intervals for *S. venezuelae* and *M. smegmatis* or in 10 min intervals for *E. coli* for 24–72 h (dependent on the bacterial species) in a final volume of 300 μl using a Bioscreen C instrument (Growth Curves US). The growth rate (OD600/min) was estimated from the slope of the linearly fitted correlation in the log-phase. The percentage of growth inhibition was calculated by comparing the log-phase growth rates in the presence and absence of the particular amsacrine derivative or the equal volume of DMSO as the control. The IC50 was calculated for each species as the concentration of a particular compound that inhibits the cell growth rate in the log-phase by 50%.

### Time-lapse microfluidic microscopy (TLMM)

For time-lapse microscopy, *M. smegmatis* strains were cultured to the log-phase of growth, then diluted with 7H9 medium supplemented with 10% OADC and 0.05% Tween-80 and loaded into the flow chamber of B04A plates with a CellASIC ONIX flow control system (Merck-Millipore). During the experiment, the cells were continuously supplied with fresh medium supplemented with the inhibitor (at 30–240 μM concentrations) or 1% DMSO in the control experiment. To visualize dead cells, after 15 h of inhibitor treatment cells were washed with propidium iodide at concentration 10 μg/ml for 1 h. The images were taken in 10 min intervals using a Delta Vision Elite Imager equipped with DV Elite CoolSnap HQ2 Camera and SoftWorx software and were subsequently analyzed using ImageJ Fiji suite (http://fiji.sc/Fiji) and R suite (http://www.R-project.org). The analysis of cell cycle and evaluation of particular phases of growth started from the moment of appearance of a single DnaN-EGFP foci (initiation of DNA replication).

## Results

### Amsacrine and its derivatives inhibit *Ms*TopA activity *in vitro*

**Figure 1:**
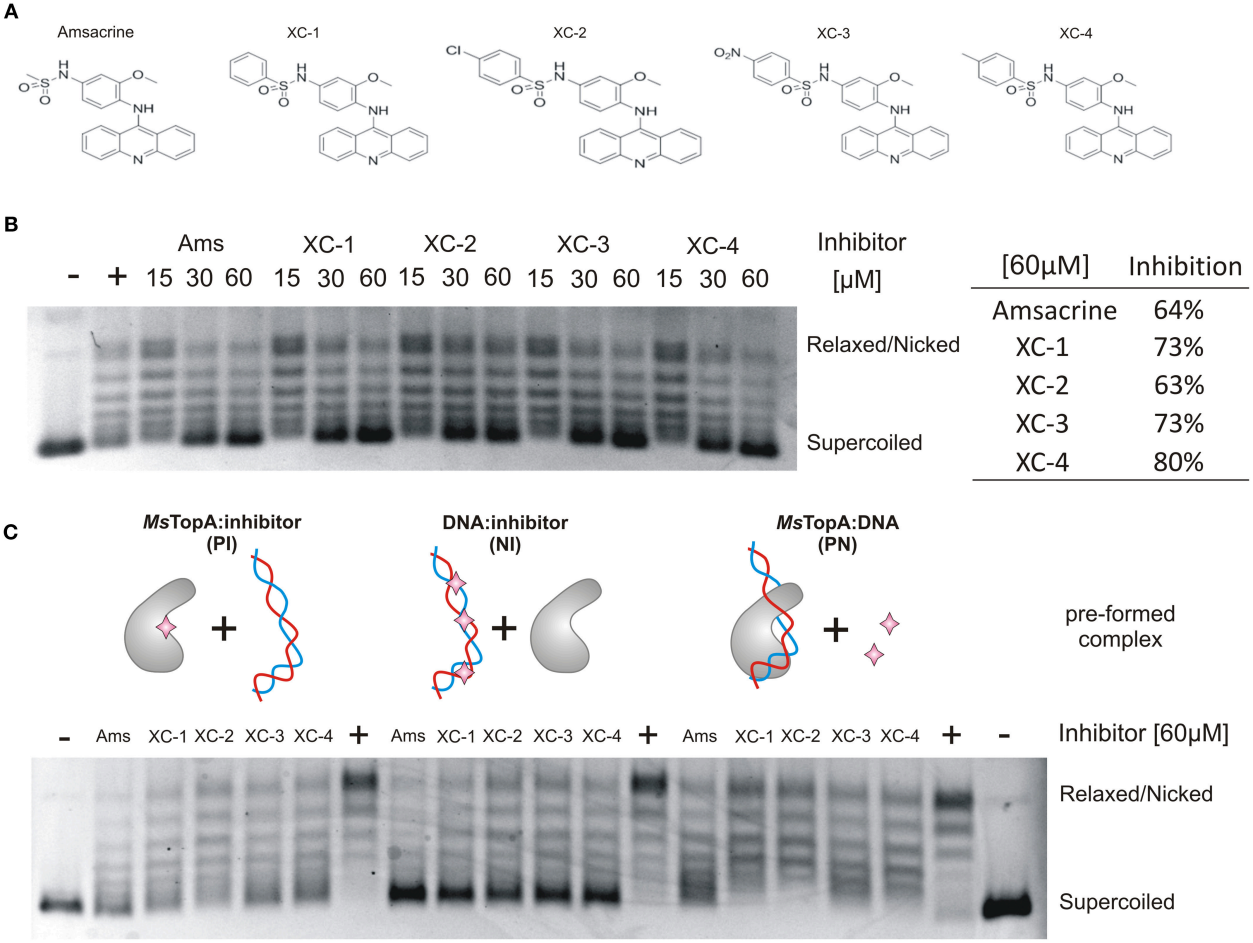
*In vitro* inhibition of *Ms*TopA topoisomerase activity and DNA binding by amsacrine or its derivatives. **(A)** The structures of amsacrine and four of its derivatives: XC-1, XC-2, XC-3, and XC-4. **(B)** Topoisomerase I activity (60 nM *Ms*TopA) in the presence of increasing concentrations (15, 30, and 60 μM) of amsacrine, XC-1, XC-2, XC-3, and XC-4. Lane “+” is the positive control of *Ms*TopA (60 nM) reaction in the presence 1% DMSO (without inhibitor added), lane “–“ is the supercoiled pUC19 plasmid in the absence of *Ms*TopA with 1% DMSO (the negative control). The increased amount of the supercoiled form of pUC19 plasmid corresponds to *Ms*TopA inhibition. The table on the right shows the inhibition of *Ms*TopA activity in the presence of 60 μM inhibitor. **(C)** The *Ms*TopA (60 nM) inhibition in protein:inhibitor (PI), DNA:inhibitor (NI), or protein:DNA (PN) assays, in which the particular complex (PI, NI, or PN) was preformed prior to adding the inhibitor (60 μM).

Amsacrine is a well-known DNA intercalating agent that interacts with DNA double helix via its acridine ring, whereas its sulfonamide side chain binds to eukaryotic type II topoisomerase (Zwelling et al., 1992). Since amsacrine was shown to inhibit topoisomerase I, according to the mechanism proposed earlier, by interacting with *Ms*TopA via the sulfonamide moiety (Godbole et al., 2014), we synthesized a set of its derivatives (Table S2) and screened this series for putative *Ms*TopA inhibitors. Using classical topoisomerase I activity assays, we compared the relaxation of negatively supercoiled pUC19 plasmid by *Ms*TopA protein (60 nM) in the presence of each tested compound (60 μM) or amsacrine as the positive control (data not shown). Among the tested compounds, only four, XC-1, XC-2, XC-3, and XC-4 (Figure 1A), efficiently inhibited *Ms*TopA activity (observed as the increase in the fraction of pUC19 plasmid that remained supercoiled). Next, the selected XC-1, XC-2, XC-3, and XC-4 compounds were analyzed in a broad range of concentrations (from 15 to 60 μM). Similar to amsacrine, they were found to strongly inhibit *Ms*TopA activity at concentrations >30 μM. The efficiency of the *Ms*TopA inhibitors at 60 μM concentration was comparable (63% TopA inhibition for XC-2) or slightly higher (73–80% inhibition for XC-1, XC-3, and XC-4) than that estimated for amsacrine (64% TopA inhibition) (Figure 1B). However, even if the concentration of inhibitor was higher (up to 90 μM, not shown), a small amount of partially relaxed topoisomers was still detectable, suggesting that the complete *Ms*TopA inhibition could not be achieved under the assay conditions. Interestingly, we did not observe *Ms*TopA inhibition by etoposide, which is also an eukaryotic topoisomerase type II inhibitor with a mode of action similar to amsacrine (Long et al., 1985; Figure S1). Our results confirm that amsacrine inhibits mycobacterial type I topoisomerases; nevertheless, its derivatives may exhibit increased efficiency of *Ms*TopA inhibition.

### Amsacrine and its derivatives decrease TopA binding to DNA

Previously, amsacrine has been suggested to inhibit *M. tuberculosis* TopA *in vitro* more efficiently when it binds to the enzyme or DNA rather than to the preformed *Mt*TopA-DNA complex (Godbole et al., 2014). Thus, using homologous *M. smegmatis* TopA (81% identity), we investigated whether such a phenomenon could also be observed for amsacrine derivatives.

Topoisomerase I activity assays showed that, indeed, the inhibitory effect of all selected compounds, including amsacrine, was the strongest when the *Ms*TopA inhibitor was pre-incubated with DNA (NI assay), which is consistent with the DNA intercalation or when it was pre-incubacted with the enzyme (PI assay), which may suggest the direct interaction between inhibitors and TopA. As observed for amsacrine, if its derivatives were added to the preformed *Ms*TopA-DNA complex (PN assay), the inhibitory effect was notably weaker, especially for XC-1 and XC-2 (Figure 1C). This observation suggested that, the formation of the *Ms*TopA-DNA complex somehow interferes with the inhibitor binding. Next, we used surface plasmon resonance (SPR) to evaluate the binding of the protein-inhibitor complex to double-stranded linear DNA fragments (241 bp) immobilized on a chip (Figure S2). As observed in topoisomerase activity assays addition of any compounds selected for testing XC-1, XC-2, XC-3, XC-4 or amsacrine (15–90 μM) to *Ms*TopA (60 nM) led to a decrease in enzyme affinity for DNA, reinforcing the idea of direct interaction between TopA and inhibitors

Overall, we confirmed that amsacrine inhibits the activity of *Ms*TopA *in vitro* presumably not only by DNA intercalation but also by preventing the formation of the enzyme-DNA complex. Moreover, we also showed that the selected amscarine derivatives exhibit similar inhibition mechanism as amsacrine.

### Amsacrine derivatives are selective inhibitors of *M. smegmatis* growth

**Figure 2:**
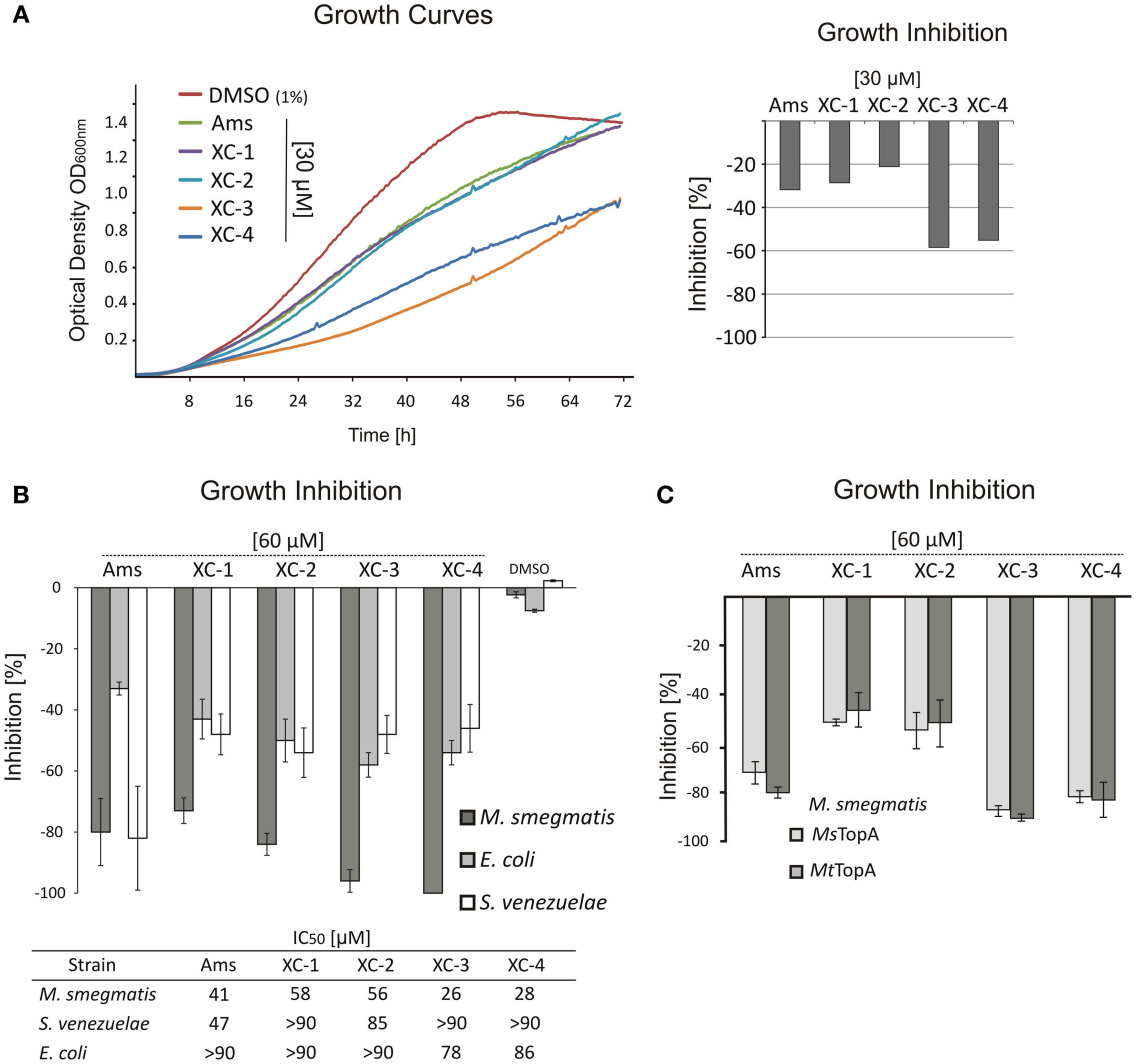
The influence of TopA inhibitors on *M. smegmatis* growth. **(A)** Growth curves of *M. smegmatis* in the presence of 1% DMSO or 30 μM amsacrine and four of its derivatives: XC-1, XC-2, XC-3, and XC-4. The diagram on the right shows the growth inhibition calculated as the difference of the growth rate at presence of 30 μM inhibitors in relation to *M. smegmatis* growth rate in the control experiment (1% DMSO, estimated as 100%). **(B)** Comparison of the growth inhibition (at 60 μM inhibitor) of three different bacterial species: *M. smegmatis, E. coli* and *S. venezuelae*. The compound concentration that inhibits growth rate by 50% (IC50) is shown in the table below the plot. **(C)** Comparison of the growth inhibition of the wild-type *M. smegmatis* (*Ms*TopA) and MSZ2 strain producing *M. tuberculosis Mt*TopA instead of the native protein.

Having proved that amsacrine derivatives inhibit DNA relaxation by *M. smegmatis* TopA *in vitro*, we tested whether the selected compounds impair the growth of wild-type *M. smegmatis* mc2155 strain. To estimate the inhibitory effect, we compared the log-phase growth in the presence of XC-1, XC-2, XC-3 XC-4, amsacrine and 1% DMSO (as controls). The strongest growth retardation was observed for the XC-3 and XC-4 compounds (55–60% growth inhibition at a concentration of 30 μM, Figure 2A), whereas the effect of amsacrine as well as the XC-1 and XC-2 derivatives was weaker (only 20–30% growth inhibition at a concentration of 30 μM). Increasing the concentration of amsacrine or its derivatives (60–90 μM) decreased the growth rate; at concentrations greater than 90 μM, each tested compound inhibited cell growth completely. Interestingly, XC-3 and XC-4 remained notably more efficient than other inhibitors at all tested concentrations (Figures S3, S4). Interestingly, in the presence of human serum albumin (HSA) the inhibitory effect of amsacrine (or its derivatives) was notably weaker (data not shown), which should be considered at potential pharmacokinetics studies. To check if the effect of amsacrine and its derivative on *M. smegmatis* growth may be enhanced by increasing the cell wall permeability, we used the low concentration of isoniazid (2.5 μg/ml) or cycloserine (10 μg/ml) but we did not observe synergistic effect of the combination of the two drugs (Figure S5).

To test the specificity of the selected inhibitors for mycobacterial cells, we also investigated the effect of XC-1, XC-2, XC-3, and XC-4 compared to that of amsacrine on the growth of two other bacterial species—closely related to mycobacteria Gram-positive *Streptomyces* and non-related Gram-negative *E. coli*. The *S. venezuelae* genome encodes the SvTopA protein, which is similar to *M. smegmatis* TopA (64% identity) (Szafran et al., 2013; Donczew et al., 2016), whereas *E. coli* TopA is homologous (43% identity) to mycobacterial TopA protein only within the N-terminal catalytic domain (Tan et al., 2016). We noticed that amsacrine inhibits *S. venezuelae* growth efficiently, similar to *M. smegmatis* growth (up to 80% inhibition at 60 μM concentration; IC50 values of 41 and 47 μM, respectively Figure S4), whereas the inhibition of *E. coli* growth was relatively weaker (32% inhibition at the same concentration; IC50 value >90 μM, Figure 2B). Interestingly, the XC-1, XC-2, XC-3, and XC-4 derivatives inhibited *S. venezuelae* growth to a much lesser extent than amsacrine (at 60 μM), suggesting their increased selectivity for mycobacteria (Figure 2B).

Finally, to examine the effect of the selected compounds on *M. tuberculosis* TopA, we constructed the *M. smegmatis* MSZ2 strain, in which the native copy of the *topA* gene was deleted and the *topA* gene from *M. tuberculosis* was delivered in trans using the integrative pMV306 vector. As expected, we did not observe any differences in growth inhibition of the wild-type *M. smegmatis* or MSZ2 strain producing *Mt*TopA (Figure 2C), clearly showing that the discrete differences in the *Ms*TopA and *Mt*TopA amino acid sequences (identity 81%) do not play any significant role in TopA inhibition by XC-1, XC-2, XC-3, and XC-4 as well as by amsacrine.

In summary, our results show that the amsacrine derivatives XC-3 and XC-4 are strong inhibitors of *M. smegmatis* growth under laboratory conditions, exhibiting IC50 values approximately 30–40% lower than amsacrine. Moreover, the toxic effect on other bacterial species (*E. coli* and *S. venezuelae*) is notably decreased, suggesting that the XC-3 and XC-4 are specific for mycobacteria.

### Amsacrine and its derivatives treatment increase the number of DnaN-EGFP complexes in cells

Since treatment with the amsacrine and its derivatives should result in topological problems that are expected to affect directly or indirectly chromosome replication, we decided to analyse their influence on replisome dynamics in *M. smegmatis* cells. For this purpose, we used an *M. smegmatis* strain (JH01) in which the beta subunit of DNA polymerase III (DnaN) was C-terminally fused to EGFP (Trojanowski et al., 2015) and analyzed its localization during the cell cycle using time-lapse microfluidic microscopy (TLMM).

**Figure 3:**
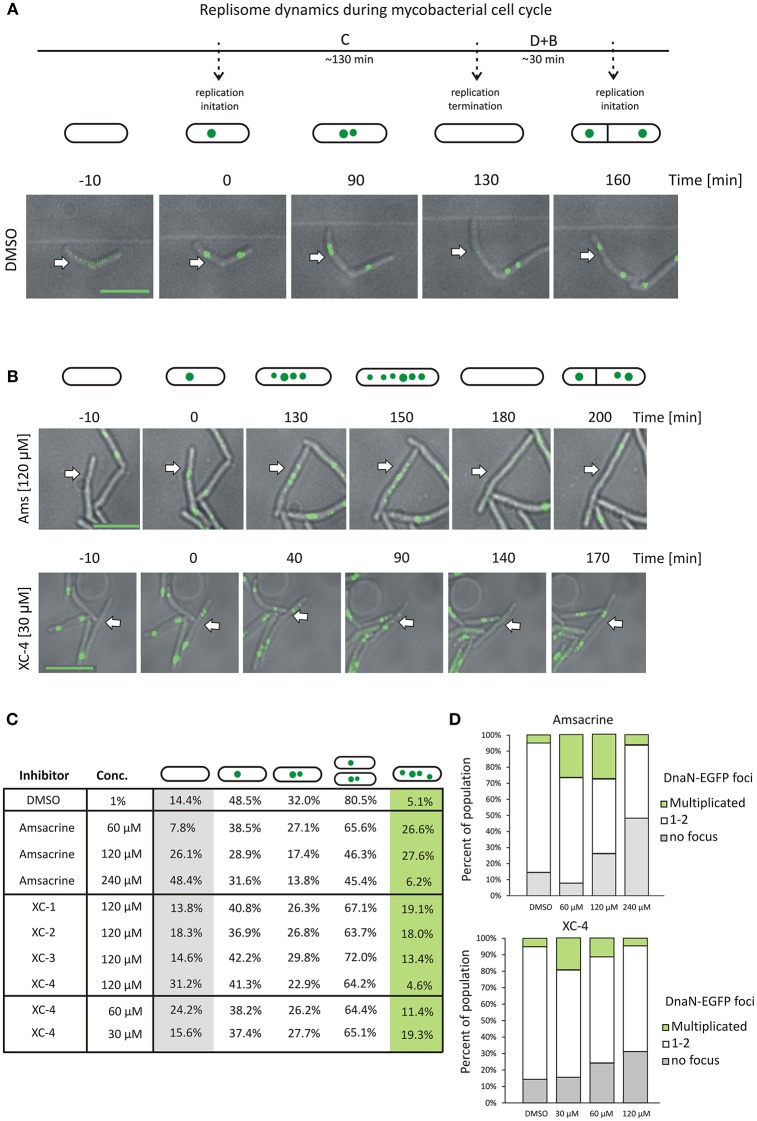
The influence of *Ms*TopA inhibition on the number of DnaN-EGFP complexes. **(A)** Canonical cell cycle of *M. smegmatis* visualized by TLMM. Phase C corresponds to chromosome replication. It starts with the appearance of a single DnaN-EGFP focus (time 0) (green dot), which subsequently splits into two foci. Phase C terminates after 130 min with the dismantling of the replisome complex. Phase D+B (30 min) is the time between the termination of DNA replication and cell division and the time after cell division prior to initiation of replication. Scale bar, 5 μm **(B)** TLMM analysis of *M. smegmatis* treated with 120 μM amsacrine or 30 μM XC-4. Analyzed cells to be followed are marked with white arrows. Scale bar, 5 μm **(C)** The percentage of cells with a different number of DnaN-EGFP foci (none, one, two or more than two) in *M. smegmatis* cultures treated with different concentrations (30–240 μM) of amsacrine or its derivatives (XC-1, XC-2, XC-3, and XC-4). The number of cells used for statistics varies from 300 to 400. **(D)** The influence of increasing concentration of amsacrine and XC-4 on the number of DnaN-EGFP complexes.

As described earlier (Trojanowski et al., 2015), during canonical growth (in the absence of a TopA inhibitor, control experiment), most of *M. smegmatis* cells had a single DnaN-EGFP focus, which subsequently split into two dynamic foci positioned close to the mid-cell, marking the ongoing chromosome replication. Shortly before cell division, the DnaN-EGFP complexes disappeared, indicating replication termination (Figure 3A). In cells treated with increasing concentrations of amsacrine (60 and 120 μM), the fraction of cells lacking a DnaN-EGFP focus (non-replicating) increased notably (from 14.4% in control experiment; see Supplementary Video 4 up to 26.1% for cells treated with 120 μM amsacrine, see Supplementary Video 1) (Figures 3B,C). In order to test viability of those cells, we performed the propidium iodide staining, which indicated that the fraction of dead cells was 12% after treatment with 120 μM amsacrine (Figure S6). This showed that about half population of the cells without replisomes (26.1 % cells for 120 μM amsacrine) are still alive, which corresponds to 14.4% of non-replicating cells in control experiments (Figures 3B,C). Moreover, the 120 μM amsacrine treatment also resulted in an increased fraction of cells (up to 27.6%) in which more than two DnaN-EGFP complexes were observed (Figures 3B–D). Remarkably, at the highest amsacrine concentration (240 μM), this fraction decreased to a similar value as in the control experiment (6.2% at 240 μM amsacrine and 5.1% in the control experiment), whereas the fraction of cells lacking replisomes reached 48%. This observation suggests that, while moderate concentrations of amsacrine promote the formation of multiple DnaN-EGFP foci, the highest concentration results in decreased cell viability, as shown earlier by Godbole et al. (2014).

The influence of 120 μM XC-1, XC-2, and XC-3 on the number of DnaN-EGFP complexes in cells was less pronounced than that observed for amsacrine. The percent of cells lacking a DnaN-EGFP focus was similar to that observed in the control experiment (13.8–18.3% for the amsacrine derivatives and 14.4% for DMSO); however, the fraction of cells with multiple DnaN-EGFP complexes increased compared to the control experiment (13.4–19.1% for XC-1, XC-2, and XC-3 and 5.1% in the control experiment) but was lower than that observed for amsacrine (Figure 3C).

The influence of 120 μM XC-4 on the number DnaN-EGFP complexes was more pronounced than the other tested inhibitors. Interestingly, treatment with 120 μM XC-4 increased the population of non-replicating cells and considerably lowered the population of cells in which multiple DnaN-EGFP complexes were observed (4.6% for 120 μM XC-4; see Supplementary Video 2 and 27.6% for 120 μM amsacrine; see Supplementary Video 1), similar to the result observed at 240 μM amsacrine (6.2%). Our *in vitro* and *in vivo* studies, suggested that that XC-4 is a stronger *Ms*TopA inhibitor than amsacrine. Thus, we expected that decreasing the XC-4 concentration (up to 30 μM; see Supplementary Video 3) should result in an increased fraction of cells with multiple DnaN-EGFP complexes, which was observed for 60 and 120 μM amsacrine. As predicted, reducing the XC-4 concentration increased the number of cells with multiple DnaN-EGFP foci (19.3 and 27.6% for 30 μM XC-4 and 120 μM amsacrine, respectively) while simultaneously decreasing the fraction of non-replicating cells (without replisomes, Figure 3D).

Overall, microscopy analysis showed that inhibition of *Ms*TopA by amsacrine and its derivatives influences chromosome replication in *M. smegmatis*. Weaker *Ms*TopA inhibition leads to an increased number of DnaN-EGFP foci in cells. Surprisingly, the highest tested concentrations of amsacrine and XC-4 result in a dramatic increase in cells lacking DnaN-EGFP complexes, reinforcing the idea that efficient *Ms*TopA inhibition leads to cell death.

### Amsacrine and its derivatives exposure elongate time of chromosome replication

**Figure 4:**
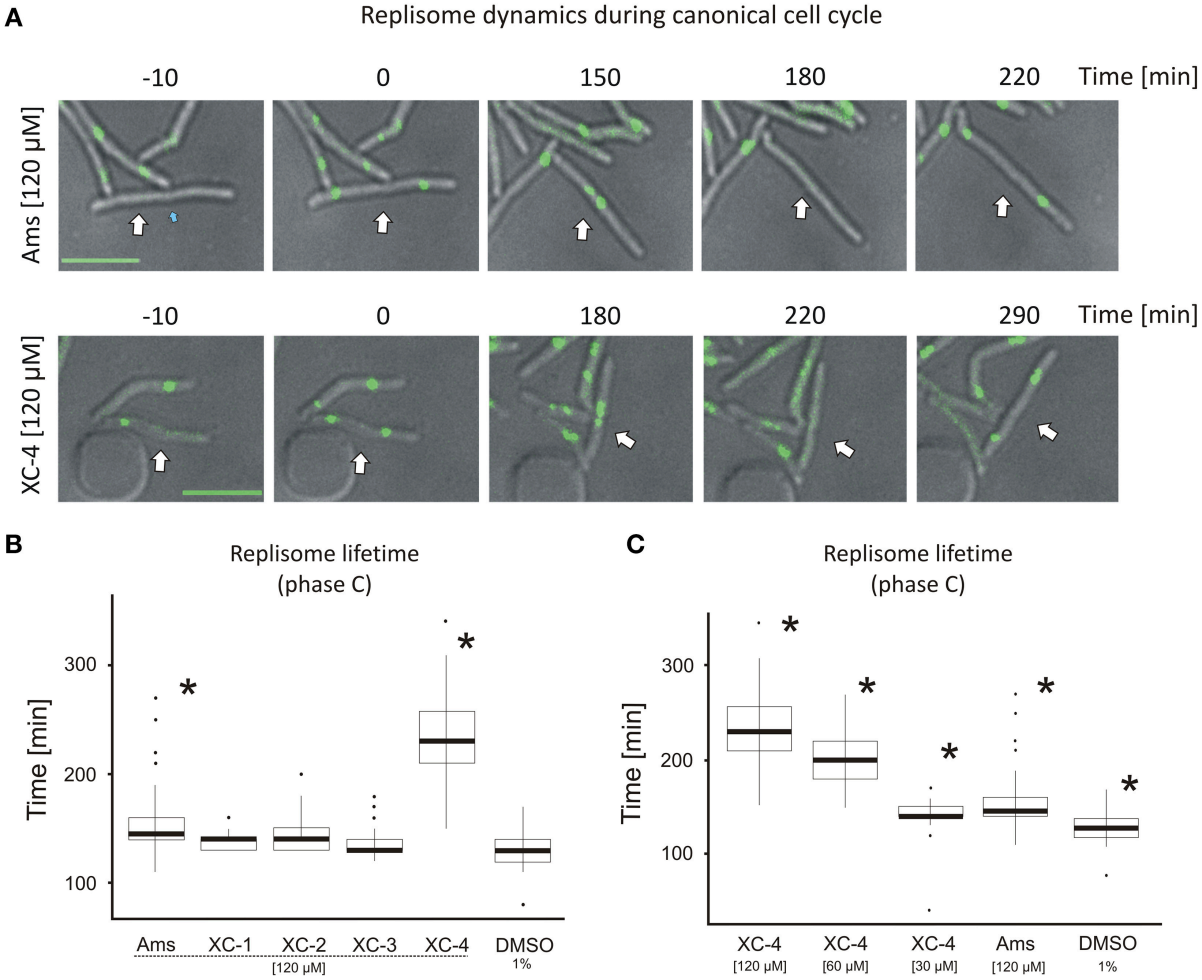
Replisome lifetime in *M. smegmatis* undergoing the canonical cell cycle **(A)** TLMM analysis of canonically replicating cells treated with 120 μM amsacrine or 120 μM XC-4 exhibiting elongated lifetime of DnaN-EGFP foci. The images show the DnaN-EGFP fluorescence (green) merged with DIC (gray). Scale bar, 5 μm. The cell followed during the time-lapse experiment was marked with white arrow. **(B)** The average lifetime of DnaN-EGFP foci (DNA replication time) in cells treated with 120 μM amsacrine or its derivatives. 1% DMSO served as the control experiment. **(C)** The average lifetime of DnaN-EGFP foci (DNA replication time) in cells treated with different concentrations of XC-4 compared to that in cells treated with 120 μM amsacrine and DMSO. The number of cells used for all statistics was 50 (asterisks indicate statistically significant *p* < 0.001 results in comparison to the DMSO treated cells).

Although amsacrine and its derivatives affected the number of DnaN-EGFP foci observed in cells, a major subpopulation of cells treated with the TopA inhibitors still showed 1-2 replisomes, as described earlier for the *M. smegmatis* cell cycle under optimal conditions (Trojanowski et al., 2015). To test whether TopA inhibitors affected the time of chromosome replication, we measured the time from DnaN-EGFP focus appearance to its disassembly (Figure 4A).

In the control experiment (in the presence of 1% DMSO), the average lifetime of the DnaN-EGFP complex was 130 min, whereas in cells treated with 120 μM XC-1, XC-2, XC-3 or amsacrine, the replisome lifetime slightly increased (Figures 4A,B). Strikingly, the presence of 120 μM XC-4 led to a significant increase in replisome lifetime up to an average of 220 min. Interestingly, the total time of cell cycle was also strongly extended for 120 μM XC-4 inhibitor, but only slightly for the same concentration of amsacrine (Figure S7). Since we previously observed that XC-4 affected *M. smegmatis* growth and number of replisomes at lower concentrations than amsacrine (Figures 2A, 3B), we investigated whether decreasing the XC-4 concentration to 30 μM would have similar effect on cells as 120 μM amsacrine. As expected, reducing the XC-4 concentration to 30 μM shortened the DnaN-EGFP lifetime, as was observed in the presence of 120 μM amsacrine (Figure 4C). This observation is consistent with the earlier results showing that treatment with 30 μM XC-4 and 120 μM amsacrine afforded similar fractions of cells showing an increased number of DnaN-EGFP complexes (19.3 and 27.6%, respectively; Figures 3A–C).

To check if that increased number of replisomes may result from decrease of TopA activity we have analyzed the localization of replisomes and *oriC* regions (using ParB-mCherry fusion, as described in Trojanowski et al., 2015) in the strain with TopA level depleted to about 25% of the wild type level (Figure S8). The TLMM analysis confirmed that TopA inhibition results in elevated fraction of cells (33% of cells in contrast to 15% in the control strain) with multiple ParB-mCherry foci, indicating increased number of chromosomes and non-canonical cell cycle. Moreover, we have observed multiple replisomes in those cells, and often reappearance of replisomes was followed by duplication of ParB-mCherry suggesting re-initiation of DNA replication, as described earlier (Trojanowski et al., 2017; Figures S8B,C). Finally the measurements of replisome lifetime showed that TopA depletion significantly affected time of replication (Figure S8C), similarly as the treatment with amsacrine or its derivatives (Figures 4B,C).

The results suggest that both amsacrine and its derivatives increase the lifetime of the DnaN-EGFP complex within the cell, indicating that inhibitors disturb the cell cycle affecting time of DNA replication. The derivative XC-4 exhibited a stronger effect on *M. smegmatis* replication lifetime than amsacrine at the same concentration, but the similar effect of lower concentrations of XC-4 and higher concentration of amsacrine suggest that mechanisms of action of both inhibitors is similar. Thus, the visualization of replisomes provides the information on the drug concentration-dependent disturbances of the cell cycle.

## Discussion

Although topoisomerase inhibitors (i.e., fluoroquinolones) are very efficient in bacterial growth inhibition, they are also poorly selective against bacterial species (Drlica, 1999). Moreover, increased resistance against routinely used antibiotics, including inhibitors of bacterial type II topoisomerase, has been reported in past few years (Bruchmann et al., 2013; Redgrave et al., 2014; Avalos et al., 2015). Thus, the search for new drugs and/or derivatives of already known inhibitors with enhanced selectivity is highly justified. Since Godbole et al. (Godbole et al., 2014) showed that amsacrine, a well-known anticancer drug targeting eukaryotic type II topoisomerase, also inhibits bacterial type I topoisomerase (TopA), we synthesized a set of amsacrine derivatives and screened them for anti-mycobacterial TopA activity. We have observed that the amsacrine and its derivatives inhibit *Ms*TopA activity, slow *M. smegmatis* growth and disturb the chromosome replication.

Among all the tested amsacrine congeners, only those with substitution of the methyl group in the sulfonamide side chain with a phenyl group (XC-1) or its chloro-, nitro- or methyl-para-substituted derivatives (the XC-2, XC-3, and XC-4 variants, respectively) retained inhibitory activity against *Ms*TopA. If the whole sulfonamide moiety was replaced by other chemical groups (Table S2) the inhibition of *Ms*TopA was abolished. This observation corroborates the earlier *in silico* prediction that the amsacrine sulfonamide side chain interacts with the Phe138, Asp111, and Ile141 residues within the TOPRIM domain (topoisomerase-primase fold) and with a proximal catalytic tyrosine residue (Tyr342) in *M. tuberculosis* TopA (Godbole et al., 2014; Tan et al., 2016; Figures S9, S10). Moreover, we noticed that the addition of a methyl group to the acridine ring also abolished the inhibition activity of amsacrine derivatives even when the sulfonamide group was retained. The acridine ring of amsacrine has been suggested to intercalate between base pairs of the DNA double helix, whereas the methoxyaniline moiety interacts with thymine groups lying in the DNA minor groove (Denny and Wakelin, 1986; Jangir et al., 2013); thus, further methylation of the acridine ring could destabilize the DNA-amsacrine complex. In *in vitro* assays, the incubation of amsacrine and its selected congeners with *Ms*TopA also affected TopA activity presumably inhibiting its interaction with DNA. Therefore, our observations support the proposed models of amsacrine-DNA and *Ms*TopA-amsacrine binding (Godbole et al., 2014).

Our *M. smegmatis* growth studies confirm the earlier observation that amsacrine efficiently inhibits Mycobacterium growth but not *E. coli* growth (Godbole et al., 2014). This may result from selective binding to *Ms*TopA. It has been proposed that the differences in the amino acid sequence in the TOPRIM domain are responsible for the selective inhibition of *M. tuberculosis* TopA by amsacrine. We also noticed that amsacrine affects the growth of *S. venezuelae* to the same extent as *M. smegmatis* growth. Since both *Streptomyces* and *Mycobacterium* belong to the phylum Actinobacteria, their TopAs are homologous (64% identity) not only in the N-terminal domain but also in the C-terminal domains rich in lysine repeats, which confers high enzyme processivity (Bhaduri et al., 1998; Szafran et al., 2014; Strzalka et al., 2017). Another possible explanation for amsacrine selectivity is based on its observed preference for AT-rich DNA sequences (Chen et al., 1988; Jangir et al., 2013). Considering that AT-rich sequences are limited in bacteria with GC-rich genomes such as Actinobacteria (Bentley et al., 2002) and that actinobacterial TopA also preferentially binds to AT-rich or single-stranded DNA (Strzalka et al., 2017), the competition between TopA and amsacrine for binding sites may be more detrimental to cell growth in Actinobacteria than in *E. coli* (50% GC). Moreover, because the amsacrine derivatives efficiently inhibit *M. smegmatis* growth but not *E. coli*, the enhanced interaction with TopA, rather than DNA intercalation (which should be increased in *E. coli* chromosomes with higher AT content) is likely to be critical for their activity.

The growth analysis suggests that TopA inhibition may lead to slower *M. smegmatis* growth. The influence of amsacrine derivatives on *M. smegmatis* growth is consistent with their TopA activity inhibition *in vitro* (Figures 1, 2). The XC-3 and XC-4 derivatives, which were the most efficient inhibitors in *in vitro* assays, were also the strongest inhibitors of culture growth. Moreover, when tested for growth inhibition, XC-3 and XC-4 exhibited higher selectivity than amsacrine for *M. smegmatis* over *S. venezuelae*. However, we did not observe any differences in the growth of an *M. smegmatis* strain expressing *M. tuberculosis* TopA (MSZ2 strain) instead of the native *Ms*TopA and the wild-type strain, confirming that the XC-3 and XC-4 derivatives also inhibit *Mt*TopA *in vivo* and excluding that the differences between *Mt*TopA and *Ms*TopA (81% identity) could interfere with the effectiveness of enzyme inhibition. The observed selectivity of XC-3 and XC-4 for mycobacterial TopAs can be possibly explained by the virtual docking of amsacrine to *M. tuberculosis* TopA (Godbole et al., 2014), where the sulfonamide group interacts with Asp111 and Ile141 in the conserved TOPRIM region of TopA. Interestingly, in both *M. tuberculosis* and *M. smegmatis* TopA, Asp111 is followed by a neutral and flexible glycine residue, whereas in *Streptomyces* TopA, it is followed by significantly larger and negatively charged glutamic acid residue (Figure S9). This may account for the difference in inhibition by amsacrine derivatives—although amsacrine may fit well to its binding site in both species, the phenyl group in its derivative XC-4 is allowed only in the binding pocket in *M. smegmatis* TopA, resulting in its specific anti-mycobacterial specificity. However, the additional explanation cannot be excluded is that modifying amsacrine increases the affinity for other unknown cellular targets present in *Mycobacterium* but not in *Streptomyces*. Nevertheless, we demonstrated that the phenyl-substituted amsacrine derivatives exhibit more efficient and selective mycobacterial TopA inhibition than amsacrine.

Visualization of replisomes showed that amsacrine or its derivatives impair chromosome replication. The observed disturbances of DNA replication are likely to be the direct effect of inhibition of TopA activity, which affects the global supercoiling of the chromosome. However, we cannot exclude that acting as interacalators amsacrine and its derivatives disturb the DNA replication by affecting any component of chromosome replication machinery. Our observations of TopA depleted strain confirmed that lowering the *Ms*TopA level affects the localization and number EGFP-fused DnaN complexes, promotes multifork replication and elongates the replisome lifetime in *M. smegmatis*. This suggests that observed aberrations of cell cycle result directly from TopA inhibition.

The lowest tested concentration of amsacrine and its most efficient derivative XC-4 led to an increased number of cells with multiple DnaN-EGFP foci. This observation can be explained by inhibition of *Ms*TopA activity. Recently, TopA has been shown to be involved in the removal of DNA-RNA hybrids that accumulate during transcription in *E. coli*. Excess hybrids lead to destabilization of the DNA double helix and may promote uncontrolled initiation of DNA replication (Baaklini et al., 2008; Martel et al., 2015). Thus, the increased number of DnaN-EGFP foci could be the result of reinitiation of DNA replication outside the *oriC* region because of the accumulation of R-loops. It also has been demonstrated that DnaN plays a role in DNA repair (Yang and Miller, 2008; Lenhart et al., 2013) thus, an additional hypothesis, that *Ms*TopA inhibition may lead to DNA damage and the induction of DnaN-dependent DNA repair, cannot be rejected. However, the results showing that DNA-RNA hybrids (which are initiation sites for DNA replication) are 5-fold more efficient in replisome loading than DNA-DNA hybrids functioning as a substrate for DNA repair (Park and O'Donnell, 2009), suggests that TopA inhibition induces re-initiation of DNA replication. The analysis of TopA depletion strain which showed the increased number of ParB-mCherry complexes correlating with the higher number of DnaN-EGFP foci suggesting that disturbances of chromosome replication induced by amsacrine derivatives directly result from TopA inhibition (Figure S8).

The highest tested concentration of amsacrine and XC-4 resulted in an increase in cells that did not show DnaN-EGFP foci. This fraction of cells consists of both living but non-replicating cells and dead cells, reinforcing the hypothesis that amsacrine has a bactericidal effect on *M. smegmatis* [16]. The extended time of replication may suggest that changes in chromosome supercoiling resulting from efficient *Ms*TopA inhibition may affect the length of all stages of *M. smegmatis* cell cycle. Moreover, during treatment with XC-4 (but not XC-3, which had a notably weaker effect than XC-4, probably due to its poor solubility under microfluidic conditions), similarly as in TopA depleted strain, replisome lifetime corresponding to replication time was extended, resulting in extension of cell cycle (Figure S8). Elongated replication time may be explained by either slower progression of replication due to topological problems or changes in gene expression. *Ms*TopA activity has been shown to regulate the expression of a large number of genes. In *E. coli*, transcription of the *dnaA* and *dnaN* genes is regulated by changes in DNA supercoiling (Peter et al., 2004); thus, it cannot be excluded that blockage of DNA replication results from changes in global transcription rather than direct involvement of the *Ms*TopA in replisome assembly or its subsequent movement along the DNA double helix.

**Figure 5:**
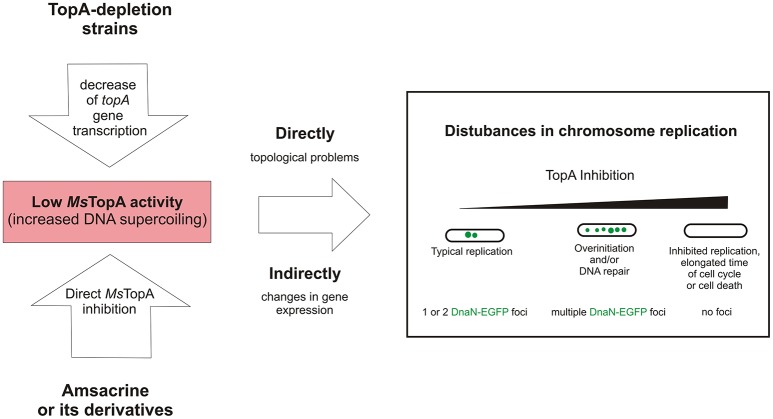
The influence of decreased *Ms*TopA activity on chromosome replication and the number of DnaN-EGFP complexes (green dots).

Taken together, our data confirm that TopA is a valuable target for anti-mycobacterial therapies and show that modification of the sulfonamide group in amsacrine increases the inhibitory activity and selectivity for mycobacterial topoisomerase I (TopA). Moreover, using TLMM, we demonstrated that amsacrine and its derivatives disturb chromosome replication in *M. smegmatis*. We suggest that the observed disturbances of chromosome replication result from inhibition of TopA activity, and this notion is supported by the observed aberrant replication in TopA depletion strain (Figure 5). Thus, we established that replisome-labeled strain may be a may be a useful tool for examining the biological effects of drugs that target topoisomerase I.

## Author contributions

MS, MK, and PS designed and performed research. BM: amsacrine derivatives synthesis; AD: expression and purification of *Ms*TopA protein; DT: MK supervision; DS: amsacrine derivatives synthesis and BM supervision; MS, and JZ-C: funding; MS and DJ wrote the paper. JZ-C: critical revision.

### Conflict of interest statement

The authors declare that the research was conducted in the absence of any commercial or financial relationships that could be construed as a potential conflict of interest.

## Supplementary material

Supplementary Video 1 JH01strain_Amsacrine_120 μM.

Supplementary Video 2 JH01strain_XC-4_120 μM.

Supplementary Video 3 JH01strain_XC-4_30 μM.

Supplementary Video 4 JH01strain_DMSO.
