# Supplementary material for: Amsacrine Derivatives Selectively Inhibit Mycobacterial Topoisomerase I (TopA), Impair M. smegmatis Growth and Disturb Chromosome Replication
Source: Front Microbiol. 2018 Jul 17;9:1592. doi: 10.3389/fmicb.2018.01592 (PMC6056748; doi:10.3389/fmicb.2018.01592)
Supplement: Supplementary file 5 [file Presentation_1.PDF]

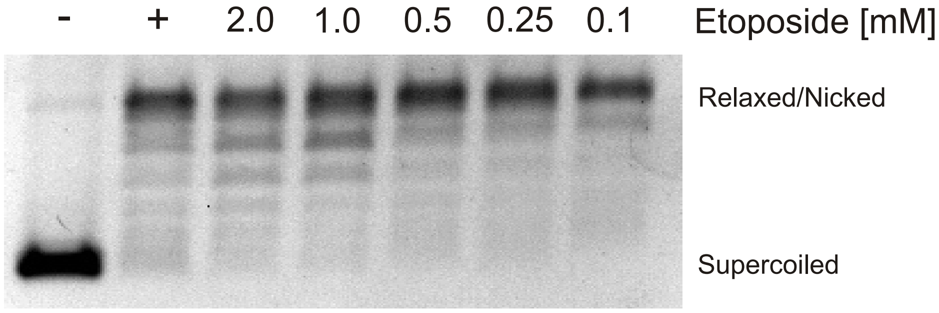

Figure S1. *MsTopA* inhibition *in vitro.* Topoisomerase I activity assay in the presence of increasing concentrations of etoposide (0.1 - 2.0 mM) and 60 nM *Ms*TopA. Lane "-" the negative control lacking *Ms*TopA, lane "+" the positive control of the reaction containing 60 nM *Ms*TopA and 1% DMSO.

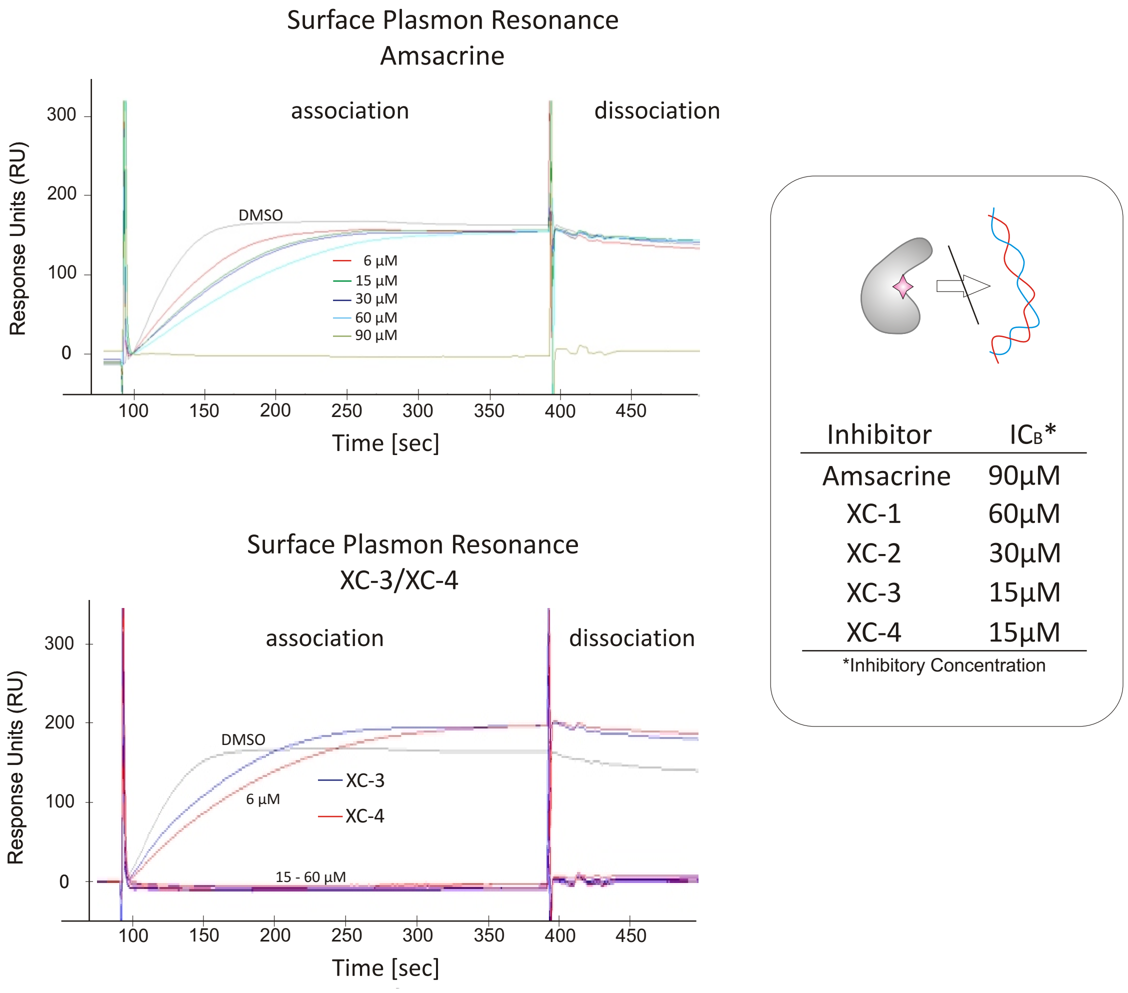

Figure S2. Analysis of *Ms*TopA-DNA binding using surface plasmon resonance (SPR). The association and dissociation curves obtained for 0-90 µM amsacrine (upper panel) and 0 - 60 µM XC-3 and XC-4 (lower panel) are shown. The inhibitory concentration that completely blocks *Ms*TopA binding (IC_B) was estimated for each compound (right panel).

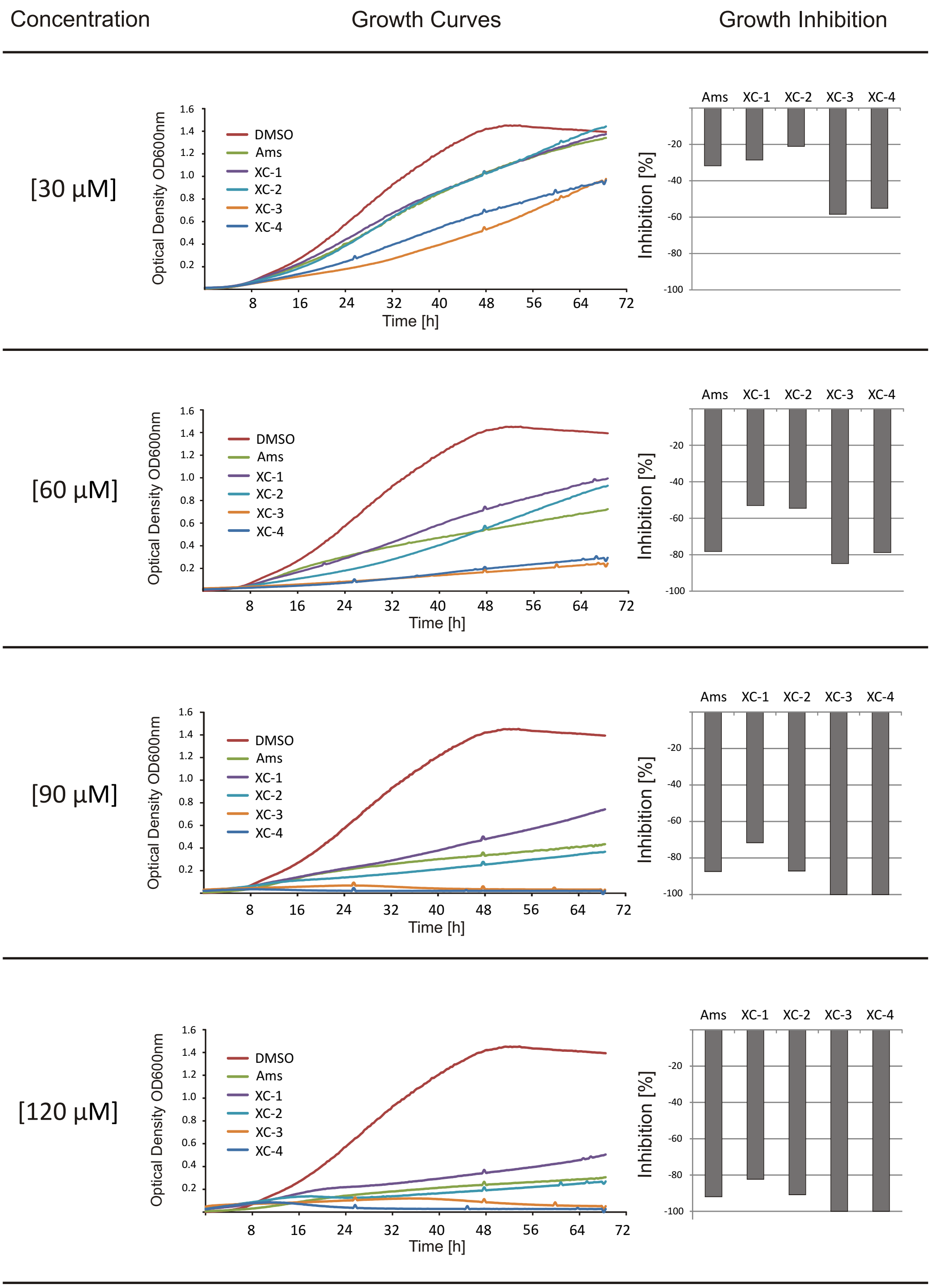

Figure S3. Analysis of *M. smegmatis* growth in the presence of *Ms*TopA inhibitors. The growth curves (each condition in triplicate) were obtained for an *M. smegmatis* strain grown in the presence of increasing concentrations of amsacrine (Ams) or its derivatives (XC-, XC-2, XC-3 and XC-4); cells treated with 1% DMSO served as the reference sample. The slope of the growth curve in the log-phase was used to estimate the growth inhibition (shown in the right panels) as follows: inhibition = $-(slope_{inhibitor}/slope_{DMSO}) * 100\%$.

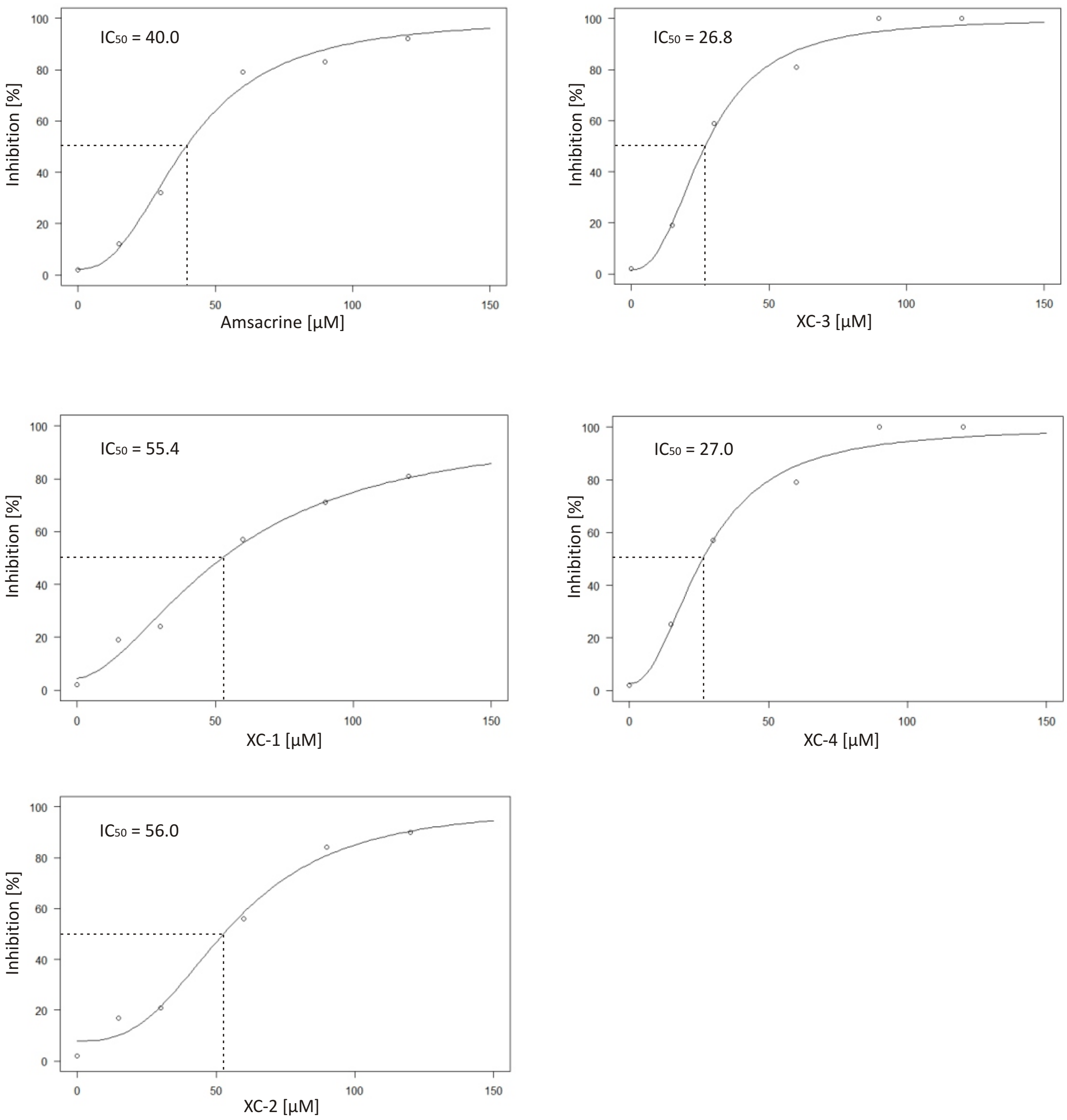

Figure S4. Calculation of the inhibitory concentration (IC_{50}). Percent of growth inhibition [%] was plotted against inhibitor concentration [µM] and fitted to a sigmoidal model using R software. Subsequently, the concentration that inhibits cell growth by 50% (IC_{50}) was estimated for each compound.

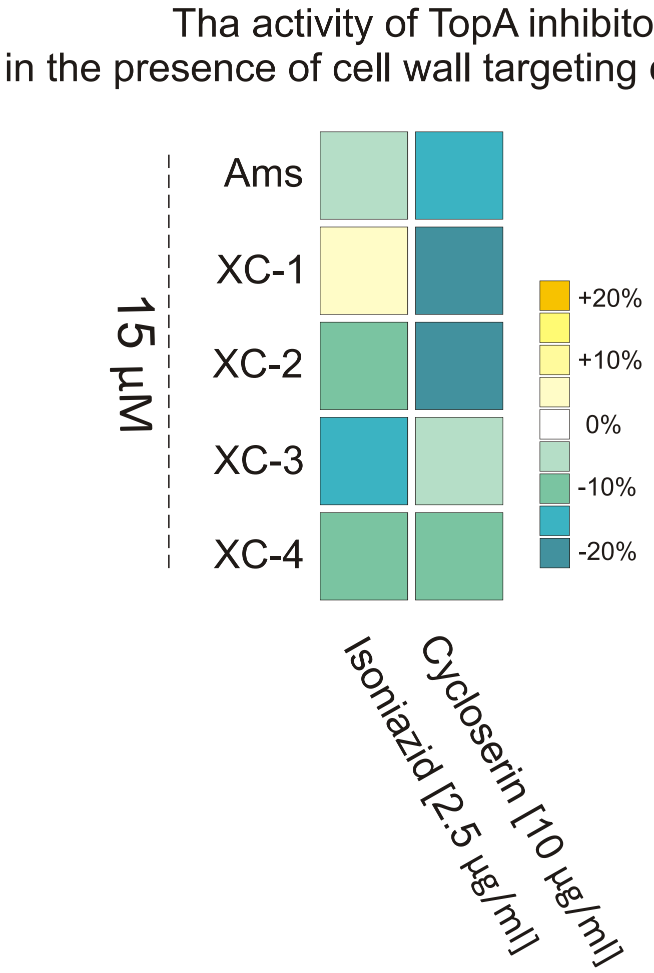

Figure S5. The growth inhibition in the presence of TopA inhibitors and cell wall targeting antimycobacterial antibiotics (isoniazid, cycloserine). To calculate the possible synergistic effect of amsacrine (Ams) or its derivatives (XC-1, XC-2, XC-3 and XC-4) and cell wall targeting compunds (isoniazid, cycloserine) we determined the growth rate of *M. smegmatis* strain using Bioscreen C (see materials and methods). We compared the growth inhibition in presence of particular drugs added separately and compared with the growth rate of *M. smegmatis* in the presence of both compounds. The results are presented as the % of increase/decrease of growth rate inhibition compared to the sum of growth rate inhibiton resulting from both compounds added separately (100%).

DMSO 120 µM Amsacrine

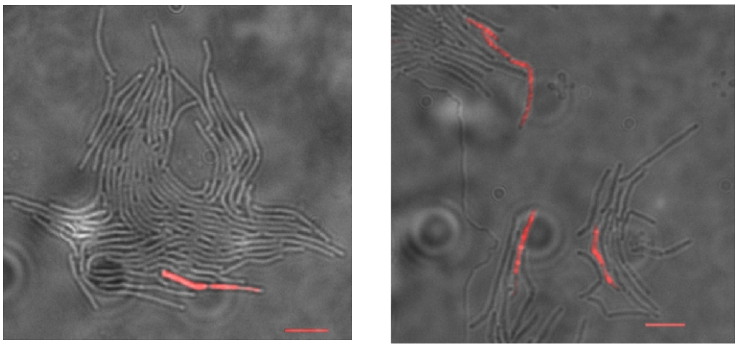

1%	12%	percent of cell [%]
n = 628	n = 512	number of cells

Figure S6. The influence of *Ms*TopA inhibition by amsacrine on *M. smegmatis* viability. *M. smegmatis* (JH01 strain) growth in the presence of 120 µM amsacrine or 1% DMSO was visualized using TLMM microscopy (the procedure descirbed in the materials and methods). After 15 hours of exposure to antibiotic cells were washed with medium suplemented with 10 µg/ml propidium iodide for 1 hour to detect dead cells (red) in the population. The percent of dead cells as well as the total number of cells (n) used for statistical analysis are shown.

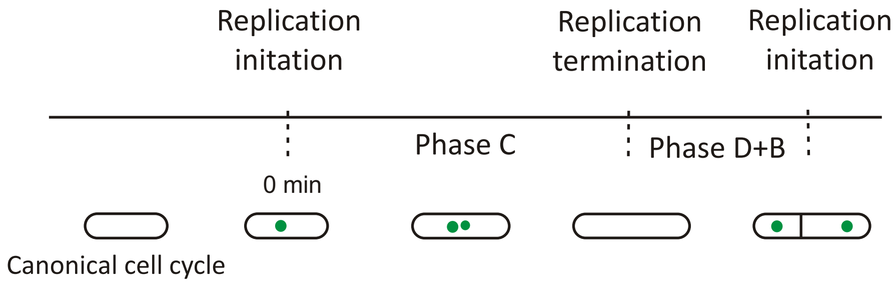

Inhibitor	Complete cell cycle [min]	Phase D+B [min]	Phase C [min]
1% DMSO	162 (18)	33 (15)	129 (15)
120 µM Amsacrine	194 (36)	40 (21)	154 (32)
120 µM XC-4	310 (60)	70 (38)	240 (51)

Figure S7. The calculation of an average time of particular phases of *M. smegmatis* cell cycle. Phase C corresponds to chromosome replication. It starts with the appearance of a single DnaN-EGFP focus (time 0) (green dot), which subsequently splits into two foci. Phase C terminates after 130 minutes with the dismantling of the replisome complex. Phase D+B (30 minutes) is the time between the termination of DNA replication and cell division and the time after cell division prior to initiation of replication. The time of complete cell cycle is the sum of C and D+B phases.The data in the table show the average time of each phase as well as the time of complete *M. smegmatis* cell cycle in TLMM experiment (see the material and methods) in control experiment (DMSO) or during treatment with amsacrine/XC-4 inhibitor. The total number of cell used for each analys is n = 50. Standard deviations are shown in brackets

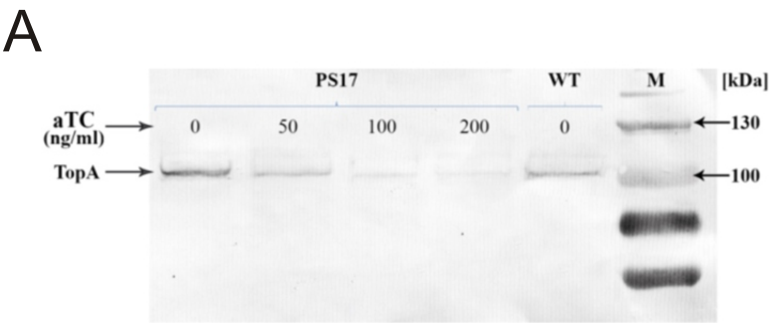

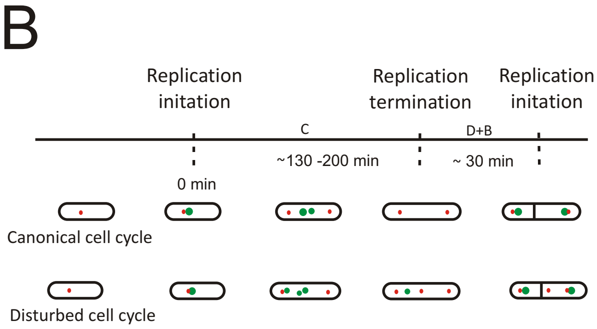

Strain	Canonical cell cycle [%]	Distrubed cell cycle [%]
TopA depletion	67%	33%
Reference	85%	15%

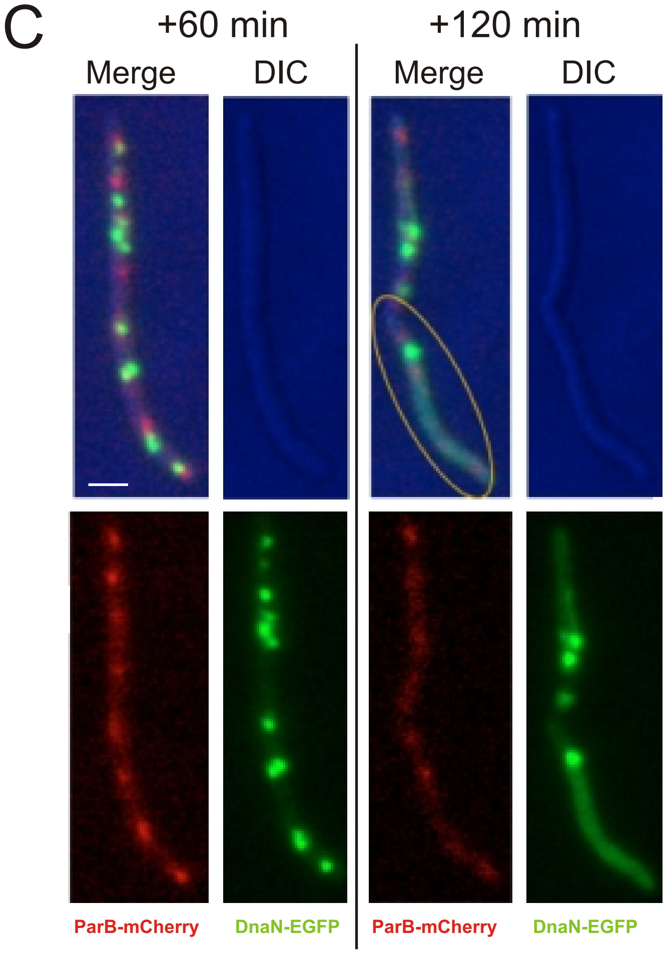

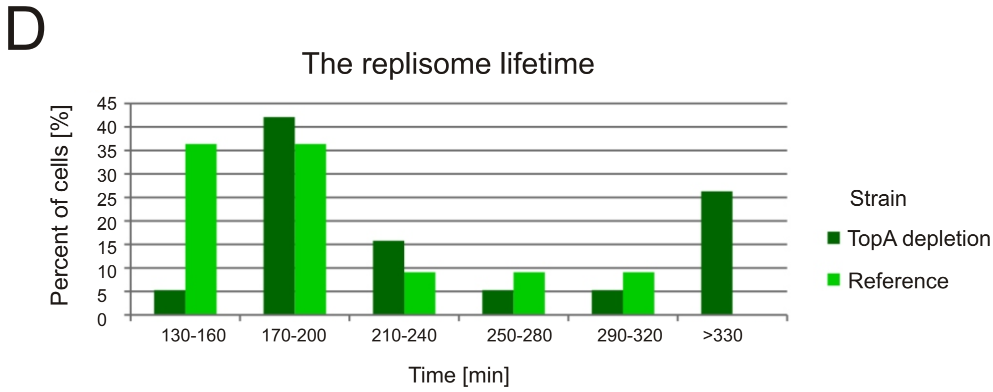

Figure S8. **The effect of *Ms*TopA depletion on DNA replication and segregation in *M. smegmatis.*** (**A**) Western blot analysis of *Ms*TopA level in TopA depletion strain (PS17) using anti-TopA polyclonal antibody (**B**) Left panel: the scheme showing canonically and non-canonically replicating cells (disturbed cell cycle). Phase C, which corresponds to chromosome replication, starts with the appearance of a DnaN-EGFP focus (green dot) (time 0), which often split into two foci. Phase C terminates after 130-200 minutes with the dismantling of the replisome complex. The number of ParB-mCherry complexes (red dots) during the *M. smegmatis* cell cycle were used to divide cells into two groups: canonically replicating cells with two ParB-mCherry complexes after completion of C phase and non-canonically replicating cells (disturbed cell cycle) with more than two ParB-mCherry complexes at the end of C phase and increased number of replisomes complexes (more than two DnaN-EGFP complexes). The percent of cells replicating canonically as well as showing disturbed cell cycle in TopA depletion strain (PS17) and the reference strain (PS29) are shown in the table below. Right panel: TLMM analysis of *M. smegmatis* strain (PS17, TopA depletion strain) producing DnaN-EGFP (green) and ParB-mCherry (red) showing cells in the C phase (60 and 120 minutes after initiation of chromosome replication) with multiple replisome and multiple ParB-mCherry foci. A single cell was marked in the circle. The depletion of *Ms*TopA level in PS17 strain (compared to the wild type strain; WT) was achieved by addition of 50 ng/ml anhydrotetracycline (aTC) to the medium.Scale bar 2 μm. (**C**) The diagram showing the replisome lifetime [min] in TopA depletion strain (PS17) and the reference strain (PS29).

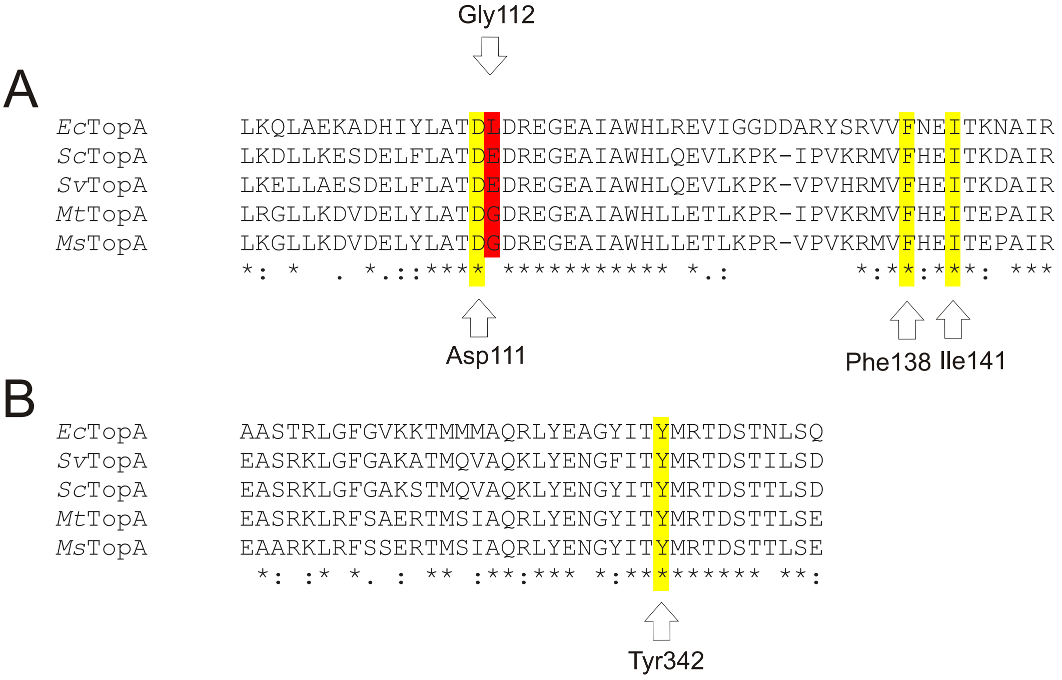

Figure S9. The alignment of TopA proteins. (**A**) The fragment of the TOPRIM domain. The conserved Asp111, Phe138 and Ile141 residues are marked in yellow. Gly112 residue, which is not present in the *S. coelicolor*, *S. venezuelae* and *E. coli* homologs of TopA, is marked in red. (**B**) The fragment of the TopA domain with the catalytic Tyr342 residue.

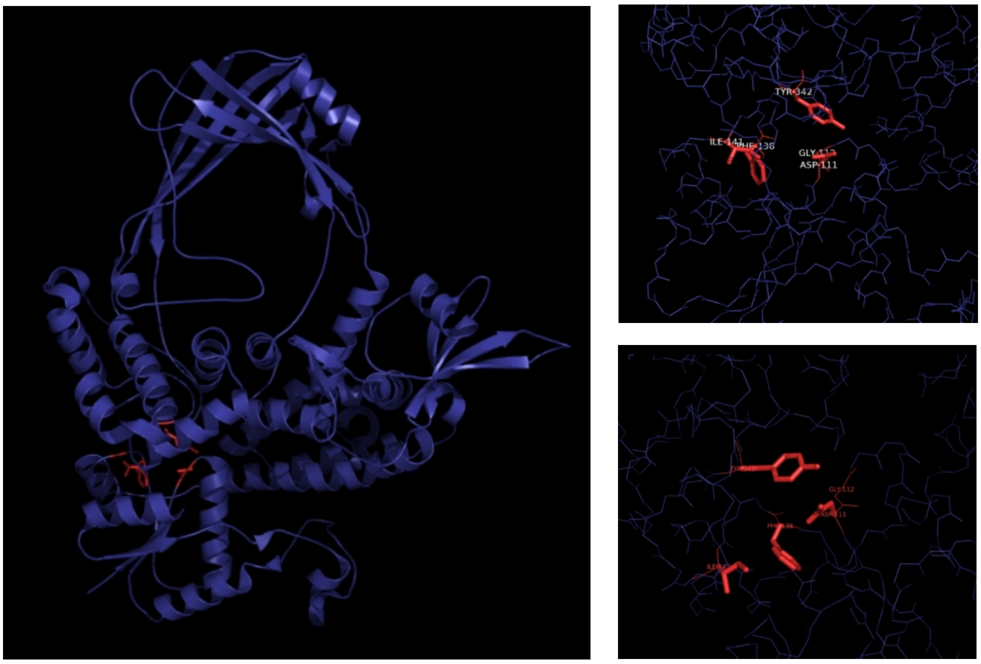

Figure S10. The positions of amino acid residues engaged in the interaction with amsacrine (according to Godbole et al. [1]) in the structure of *M. tuberculosis* TopA. The highly conserved amino acids residues crucial to the amsacrine interaction (Ile141, Phe136, Tyr342, and Asp111) as well as the poorly conserved Gly112 are marked in red. On the right is shown a zoomed in view of the TOPRIM domain in the same orientation as the whole structure (up) and rotated (down).

SUPPLEMENTARY MATERIALS AND METHODS

Bacterial strains

To construct strain producing *M. tuberculosis* MtTopA protein (MSZ2), we first created MSZ1 strain (see Table 1), in which the native copy of the Ms*topA* gene was removed and complemented with *in trans* delivered pMV306 integrating vector (hygromycin B resistant, HygR) carring Ms*topA* gene under control of its native promoter. In the first step Ms*topA* gene with its 575-base pair (bp) upstream region was amplified (using promtopAMSRV and XbaItopAMSFW oligonucleotides) and ligated to pMV306 plasmid yelding in pMV306_Ms*topA* plasmid, which was subsequently introduced to *M. smegmatis* mc2 strain (the wild type laboratory strain). In the next step the native copy of Ms*topA* gene was replaced with apramycin restistance cassette in result of homologous recombination with p2NIL_Ms*topA*::Apra vector. To create p2NIL_Ms*topA*::Apra plasmid, two DNA fragments (~1500 bp each) flanking the *topA* gene from the *M. smegmatis* chromosome were amplified (with primers topAMSFW1 and topAMStopARV2 as well as topAMSFW3 and topAMSRV4), digested with HindIII/NotI and NotI/PacI restriction enzymes, and ligated to p2NIL vector [2] previously digested with HindIII and PacI. Subsequently, the obtained vector was digested with NotI restriction enzyme, dephosphorylated and ligated with the apramycin resistance cassette, which was amplified from pIJ773 vector [3] using ApraNotIFW and ApraNotIRV oligonucleotides, purified and digested with NotI. The vector p2NIL_Ms*topA*::Apra was used for transforamtion of *M. smegmatis* pMV306_Ms*topA* strain, which were selected subsequently for for double-crossover according to the procedure described by Ginda et al. [4] using apramycin resistance for additional selection yeliding in MSZ1. Next, we substituted the pMV306_Ms*topA* in MSZ1 strain with the pMV306_Mt*topA* plasmid (kanamycin resistant, KnR) containing the *M. tuberculosis* Mt*topA*. To construct pMV306_Mt*topA* we amplified the fragment encompassing the Mt*topA* gene with its 507-bp upstream region using the promtopAMT_FW and Mt-topA_RV1 oligonucleotides and cloned subsequently into the integrative pMV306 vector (KnR). The pMV306_Mt*topA* vector was used for transformation of MSZ1 strain aaccording to procedure described by Pashley and Parish [5] and selected for kanamycin-resistant and hygromycin-sensitive colonies, obtaining MSZ2 strain.

M. smegmatis strain with the controlled expression of the Ms*topA* gene (PS17) was constructed in the background of *parB-mcherry*, *dnaN-egfp* strain PS03 [6]. First, Ms*topA* gene was excised from pET28*topA* plasmid by digestion with NdeI and HindIII and cloned to modified pKW08 plasmid [7], yielding pKW08p*tetRO**topA*. pKW08p*tetRO**topA* (HygR) was introduced to PS03 yielding strain PS05, which was further modified, by deletion of the native Ms*topA* gene, using p2NIL_Ms*topA*::Apra vector described above. The obtained strain PS07 (*parB-mcherry, dnaN-egfp* Ms*topA*::Apra pKW08p*tetRO**topA*) was used for plasmid replacement strategy [5] to deliver the strain with the tightly controlled Ms*topA* expression.
The construct for controlled Ms*topA* expression was prepared on the basis of pTC-28S15-0X vector (KnR) [8].The pTC-28S15-0X vector contains modified reverse *tetRrev* gene under the control of p*smyc* promoter. Reverse TetR binds to p*myctetO* sites in presence of tetracycline or anhydrotetracyline inhibiting expression of the gene downstream. The Ms*topA* gene was cloned to pTC-28S15-0X vector as follows: Ms*topA* was cut out from pKW08p*tetRO**topA* with PspM01 restriction enzyme together with the fragment of p*tetRO* promoter, blunt ended and subsequently ligated to EcoRV digested pTC-28S15-0X vetor. Next, the obtained plasmid was modified by introduction of p*myctetO* sites upstream of the Ms*topA* gene. p*myctetO* was used for recombination as part of apramycin resistance cassette amplified with the primer pTC28s15RecFW and pTC28s15RecRV and flanked with SnaBI restriction sites. In the next step apramycin cassette was removed by digestion with SnaBI and re-ligated leaving p*myctetO* upstream the Ms*topA* gene. pTC-28S15-0X p*smyc* *tetRrev*p*myc* Ms*topA* construct was used for transformation to PS07 strain to replace pKW08p*tetRO**topA* (HygR). The obtained strain PS17 was verified by sequencing and Southern blotting. The Ms*topA* expression in PS17, when cultured in presence of 200 ng/ml anhydrotetracycline was estimated using Western blotting to be 20-30% of the wild type level (Fig. S6). The reference strain PS29 was constructed by introduction of pTC-28S15-0X plasmid to PS03 strain.

Surface plasmon resonance (SPR)

SPR analyses were performed on a BIAcore T200. For the SPR analyses, the biotinylated fragment (241-bp long) was amplified using parA4200 and parS4440_rv_biot oligonucleotides from *S. coelicolor* chromosome. DNA immobilization on the surface of the sensor chip SA and protein binding analyses were performed according to the procedure described by Strzalka et al.[9]. Prior to binding to the DNA-coated chip, *Ms*TopA (50 nM) in 25 mM NaH_2PO_4 pH 8.0, 150 mM NaCl and 5% glycerol (binding buffer) was incubated on ice for 40 minutes with amsacrine or its XC derivatives (in 15 - 90 µM concentrations or 1% DMSO for the control experiment). For each compound, the IC_B value was estimated as the inhibitor concentration that completely abolished the binding of $_{Ms}$TopA to the DNA-coated chip.

Supplementary literature

1. Godbole AA, Ahmed W, Bhat RS, Bradley EK, Ekins S, Nagaraja V. Inhibition of Mycobacterium tuberculosis topoisomerase I by m-AMSA, a eukaryotic type II topoisomerase poison. Biochem Biophys Res Commun. 2014;446: 916920. doi:10.1016/j.bbrc.2014.03.029

2. Parish T, Stoker NG. glnE is an essential gene in Mycobacterium tuberculosis. J Bacteriol. 2000;182: 57155720.

3. Gust B, Chandra G, Jakimowicz D, Yuqing T, Bruton CJ, Chater KF. Lambda red-mediated genetic manipulation of antibiotic-producing Streptomyces. Adv Appl Microbiol. 2004;54: 107128. doi:10.1016/S0065-2164(04)54004-2

4. Ditkowski B, Holmes N, Rydzak J, Donczew M, Bezulska M, Ginda K, et al. Dynamic interplay of ParA with the polarity protein, Scy, coordinates the growth with chromosome segregation in Streptomyces coelicolor. Open Biol. 2013;3: 130006. doi:10.1098/rsob.130006

5. Pashley CA, Parish T, McAdam RA, Duncan K, Stoker NG. Gene replacement in mycobacteria by using incompatible plasmids. Appl Environ Microbiol. 2003;69: 517523.

6. Trojanowski D, Ginda K, Pióro M, Hołówka J, Skut P, Jakimowicz D, et al. Choreography of the Mycobacterium replication machinery during the cell cycle. mBio. 2015;6: e02125-02114. doi:10.1128/mBio.02125-14

7. Williams KJ, Joyce G, Robertson BD. Improved mycobacterial tetracycline inducible vectors. Plasmid. 2010;64: 6973. doi:10.1016/j.plasmid.2010.04.003

8. Klotzsche M, Ehrt S, Schnappinger D. Improved tetracycline repressors for gene silencing in mycobacteria. Nucleic Acids Res. 2009;37: 17781788. doi:10.1093/nar/gkp015

9. Strzalka A, Szafran MJ, Strick T, Jakimowicz D. C-terminal lysine repeats in Streptomyces topoisomerase I stabilize the enzyme-DNA complex and confer high enzyme processivity. Nucleic

Table S1 Oligonucleotides

Oligonucleotide	Sequence	Description
parA4200	CCGGATCCGGCTTCGCTCGGATGG	SPR
parS4440_rv_biot	ACTCCGTGCCCTCCCCGAGC	SPR
MstopANdeIFW	GGCATATGTTGGCTGGCGGCGACCG	*Ms*TopA cloning and purification
MstopABamIHRV	CCGGATCCCTAGGCCTTCTTGGCGGC	*Ms*TopA cloning and purification
promtopAMT_FW	GGTACCCGGGGGACCCGA	Construction of MSZ2
Mt-topA_RV1	GGTCTAGACTAGTCGCGCTTGGCTGCC	Construction of MSZ2
promtopAMSRV	GATATCGGTGGTGGTCGCG	Construction of MSZ2
XbaIMSFW	TTTCTAGACTAGGCCTTCTTGGCGGCG	Construction of MSZ2
topAMSFW1	TTAAGCTTGAATCCTCAACGCGTGTTCAA	Construction of MSZ2
topAMSRV2	TTGCGGCCGCTGGAGACCGTGACGCTCGA	Construction of MSZ2
topAMSFW3	TTGCGGCCGCTTAAACGAGGTAGCCAGCTATCTTG	Construction of MSZ2
topAMSRV4	TTTTAATTAACCGGACCTCCTGTCGTTC	Construction of MSZ2
ApraNotIFW	TTGCGGCCGCGGAACTTCGGAATAGGAACTT	Construction of MSZ2
ApraNotIRV	TTGCGGCCGCCAGCTCCATCAGCAAAAGGG	Construction of MSZ2
pTC28s15RecFW	TGACGGTGCCGACGATCCAATATTTCTAGTAGATCTGATTACGTAT AGGCTGGAGCTGCTTC	Construction of PS017
pTC28s15RecRV	CAGGATTCCACGATGAGAGTAGCGAGCAGTCAGATGAGACTCCCA GAGCCTATCTATCACTGATAGGGAGGACAAACTCTATCACTGATAGG GAGTTCTCCCTACGTAATGGGTTCATGTGCAGCTC	Construction of PS017

Synthesis of the amsacrine derivatives

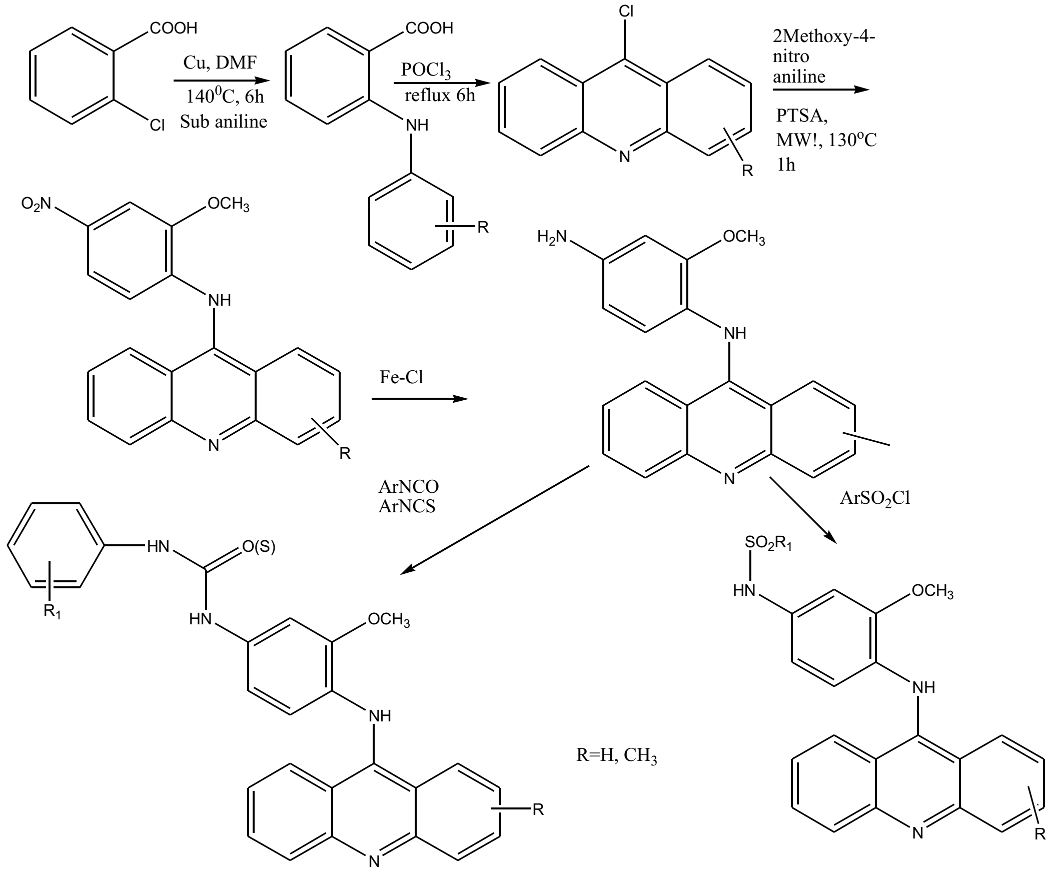

Preparation of substituted 2-(phenylamino)benzoic acid derivatives: To a stirred solution of 2-chlorobenzoic acid (10 g, 64 mmol) in DMF (N,N-dimethylformamide), potassium carbonate (13.24 g, 96 mmol), the corresponding aniline (70.4 mmol) and copper powder were added and heated up to 140 °C for 6 h. The reaction progress was monitored by TLC, and after completion, the reaction mixture was filtered through Celite, and the Celite bed was washed with ethyl acetate. The combined organic layers were evaporated under reduced pressure, and the resultant crude residue was purified by flash column chromatography using 5-20% ethyl acetate: hexane as eluent on 100-200 mesh silica gel to obtain the corresponding phenylamino benzoic acid derivatives in good yield. These intermediates were confirmed by mass (ESI mode) and used in the next step.

Preparation of 9-chloroacridine derivatives from the substituted phenylamino benzoic acid intermediates: The phenylamino benzoic acid derivatives (47 mmol) were cyclised intramolecularly to 9-chloroacridine derivatives by refluxing them in 60 ml of $POCl_3$ for 4 h, and the reaction was monitored by TLC. Excess $POCl_3$ was removed by a rotary evaporator under reduced pressure. Crushed ice was added to the resulting solution, and it was neutralized to pH ~7 with saturated bicarbonate solution. The solid was separated by filtration, dried and purified by flash column chromatography using 5-10% ethyl acetate: hexane as eluent on 60-120 mesh silica gel to obtain the corresponding 9-chloroacridine derivatives in good yield. These compounds were confirmed by mass spectrometry and used in the next step.

Preparation of N-(2-methoxy-4-nitrophenyl)acridine/2-methylacridine-9-amine intermediates: The 9-chloroacridine derivatives (10 mmol) were reacted with 4-nitro-o-anisidine (1.8 g, 10.7 mmol) under microwave conditions in methanol using PTSA (para-toluenesulfonic acid) as a catalyst (5-10 mg) at 130 °C for 1 h, and the reaction was monitored by TLC. After completion of the reaction, methanol was evaporated under vacuum, and the crude residue was dissolved in ethyl acetate and washed with saturated bicarbonate solution. The organic layer was dried over anhydrous sodium sulfate and evaporated under vacuum, and the resultant crude mixture was further purified by flash column chromatography on 60-120 silica gel using 10-35% ethyl acetate: hexane as eluent to obtain the product as a yellow solid.

Preparation of N1-(acridin/2-methylacridin-9-yl)-2-methoxybenzene-1,4-diamine intermediates: The corresponding nitro acridine derivatives (6 mmol) were converted to amine derivatives via reduction with Fe- HCl in ethanol by refluxing for 4 h. The crude reaction mixture was diluted with ethyl acetate and filtered through a Celite bed, and the organic layer was dried over sodium sulfate and concentrated under vacuum. The obtained crude mixture was purified by flash column chromatography using ethyl acetate: hexane as eluent to obtain the product as a brown solid in good yield.

Preparation of N-(3-methoxy-4-((acridin/2-methylacridin-9-yl)amino)phenyl) methane/ substituted benzene sulfonamide derivatives: For the preparation of the sulfonamide derivatives, the corresponding acridine amine derivatives (0.6 mmol) (5a-b) were dissolved in 5 ml DMF, and to this mixture, triethylamine (Et3N, 1.38 mmol) and the corresponding substituted alkyl/aryl sulfonyl chlorides (0.6 mmol) were added at 0 °C and allowed to stir at rt for 3 h. After completion of the reaction, crushed ice was added to the crude reaction mixture, and the precipitated solid was filtered, dried and further purified by flash column chromatography using 5-10% methanol: dichloromethane as eluent to obtain the sulfonamide derivatives in good yields.

Preparation of 1-(3-methoxy-4-((acridin/2-methylacridin-9-yl)amino)phenyl)-3-phenylthiourea/ urea derivatives: For the preparation of the thiourea and urea derivatives, the corresponding acridine amine derivative (0.6 mmol), Et3N (1.38 mmol) and a substituted phenyl isothiocyanate/isocyanate (0.6 mmol) were dissolved in ethanol and heated at 80 °C for 8 h. After completion of the reaction, the precipitated solid was filtered, dried and recrystallized from ethanol to obtain the final thiourea and urea derivatives.

Table S2 Amsacrine derivatives

Compound	Structure	Molecular Weight
XS-1	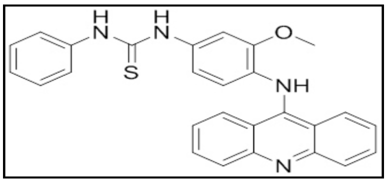	450.53
XS-2	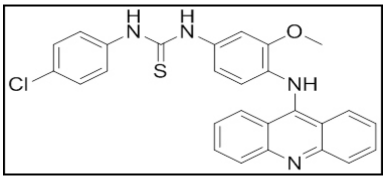	484.97
XS-3	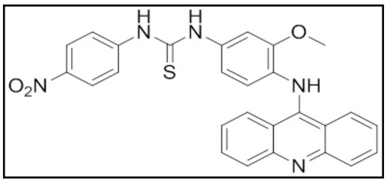	495.53
YS-1	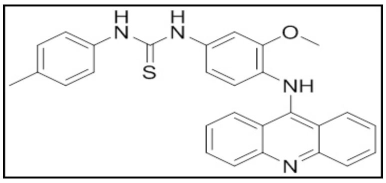	464.16
YS-2	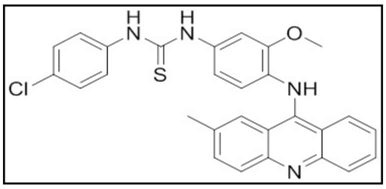	498.12
YS-3	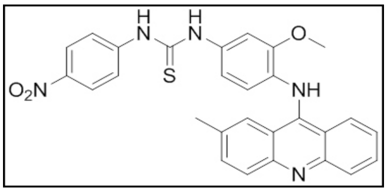	509.55
YS-4	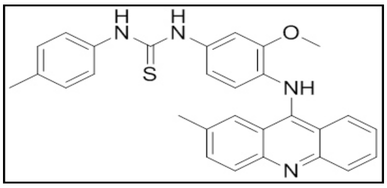	478.58
XU-1	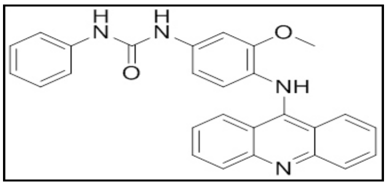	434.46

XU-2	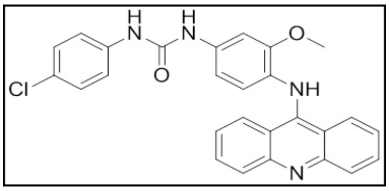	468.91
XU-3	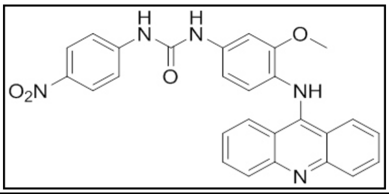	479.46
XU-4	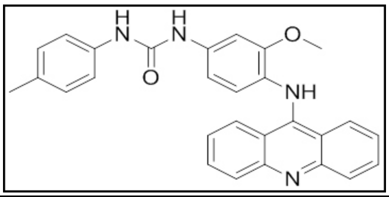	448.49
YU-1	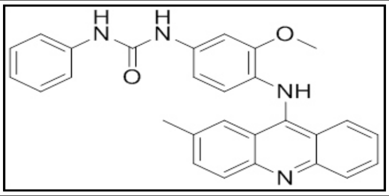	448.49
YU-2	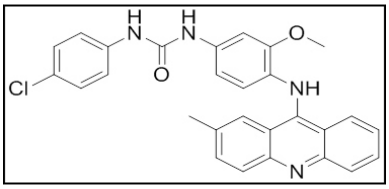	482.93
YU-3	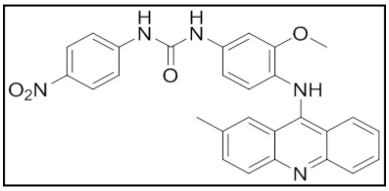	493.49
YU-4	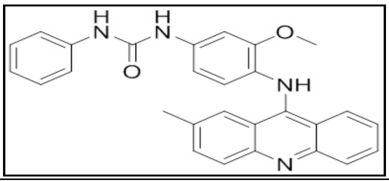	462.52
XC-1	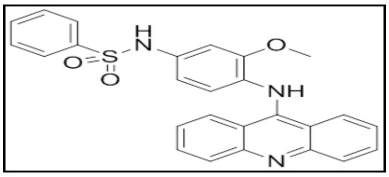	455.5
XC-2	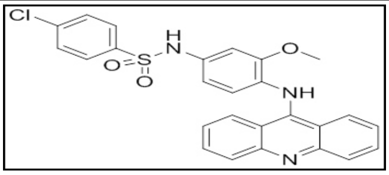	489.95

XC-3	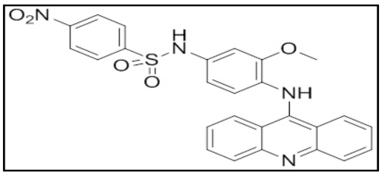	500.5
XC-4	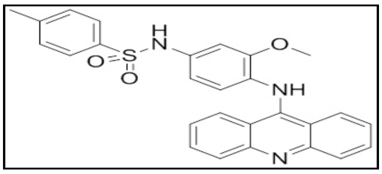	469.53
YC-1	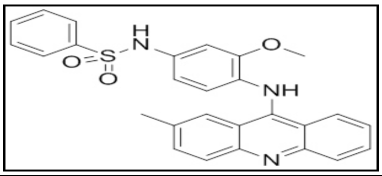	469.53
YC-2	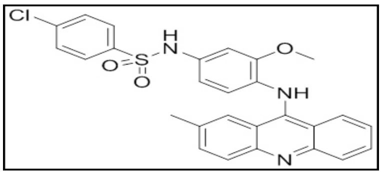	503.97
YC-3	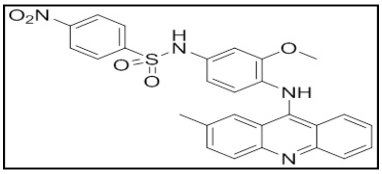	514.53
YC-4	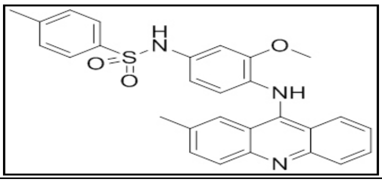	483.56
